# Predicting anti-CCP positivity and early rheumatoid arthritis onset from routine laboratory parameters: a SHAP-explained machine learning pipeline

**DOI:** 10.3389/fmed.2026.1834291

**Published:** 2026-06-29

**Authors:** Juan Wang, Jiaqing Chen, Kaiwen Wang

**Affiliations:** 1 Clinical Laboratory, Shanghai Clinical Research and Trial Center, Shanghai, China; 2 Trauma Center, Zhucheng People's Hospital, Weifang, Shandong, China; 3 Rheumatology and Immunology Laboratory, Jiading District Central Hospital Affiliated Shanghai University of Medicine & Health Sciences, Shanghai, China

**Keywords:** anti-CCP antibody, biomarkers, class imbalance, deep learning, early risk prediction, inflammatory markers, machine learning, Matthews correlation coefficient

## Abstract

Early and accurate prediction of rheumatoid arthritis (RA) is critical for improving patient prognosis; however, existing approaches rely excessively on single autoantibody markers, neglect the systematic predictive value of routine hematological parameters, and lack mechanistic interpretability. In this study, 500 patients attending a rheumatology outpatient clinic were enrolled, and 29 routine laboratory features were collected. Anti-CCP positivity and early RA onset within 12 months were defined as dual binary prediction targets. Five traditional machine learning models (logistic regression, random forest, gradient boosting, SVM, and KNN) and five deep learning models (MLP, ResNet, Transformer, AE-Classifier, and TCN) were systematically compared using six evaluation metrics: accuracy, AUC-ROC, F1-score, precision, recall, and Matthews correlation coefficient (MCC). A four-dimensional SHAP explainability analysis was subsequently applied to the best-performing deep learning model. Logistic regression achieved the best overall performance (Accuracy = 0.848, AUC = 0.857, F1 = 0.910, MCC = 0.441). Among deep learning models, the Transformer performed relatively well (AUC = 0.812), whereas ResNet and TCN exhibited severe class collapse (MCC ≈ 0). SHAP analysis identified ESR (Mean|SHAP| = 0.097) and CRP (0.090) as the most important positive predictive drivers, and albumin (ALB, 0.062) as the key protective factor, together forming a core biomarker triad for early RA risk prediction. Dependence plots further revealed the synergistic interaction between ESR and CRP, as well as a non-linear protective threshold effect of ALB. Individual waterfall plots confirmed close alignment between model decisions and clinical pathological mechanisms. The proposed machine learning pipeline based on routine laboratory parameters can effectively predict early RA risk, and the SHAP explainability analysis transforms the model “black box” into clinically readable decision rationale, providing evidence-based support for optimizing early screening strategies in rheumatology.

## 1 Introduction

Rheumatoid arthritis (RA) is a chronic systemic autoimmune disease characterized by persistent synovial inflammation (1), with a global prevalence of approximately 0.5%–1.0% and approximately 0.42% in China, affecting more than five million patients, with women approximately three times more likely to be affected than men. Without timely diagnosis and effective intervention, RA progressively leads to irreversible articular cartilage destruction, bone erosion, and loss of joint function, severely impairing patients' quality of life and imposing a substantial socioeconomic burden. The 2010 ACR/EULAR revised classification criteria established anti-cyclic citrullinated peptide (anti-CCP) antibody as the core serological marker, with a specificity as high as 95%–98%, detectable in serum years before the onset of clinical symptoms. However, approximately 30% of early RA patients present with low-titer or seronegative anti-CCP status, making it difficult to achieve systematic early identification of high-risk populations based solely on traditional antibody threshold determination. Meanwhile, routine laboratory parameters—including inflammatory markers (CRP and ESR), complete blood count-derived indices (NLR and PLR), and nutritional/metabolic indicators (ALB)—are significantly altered during the pathological process of RA and have potential value as multidimensional combined predictive biomarkers; however, their systematic quantitative associations with anti-CCP positivity and early RA onset have not yet been fully explored.

Against this background, machine learning (ML) technology, owing to its capacity to simultaneously integrate multidimensional non-linear features and adaptively learn complex interaction information, has demonstrated significant advantages in predictive modeling for cardiovascular disease (2–4), diabetes (5), cognitive impairment (6), cancer (7), and increasingly in rheumatic and musculoskeletal diseases (8–10), among other conditions. Greener et al. (11) systematically reviewed the application pathways of machine learning in biomedical research, emphasizing the inherent advantage of multi-feature joint modeling over single-marker prediction. Huang et al. (7) further confirmed the broad applicability of machine learning in survival prediction from real-world clinical data through meta-analysis. At the deep learning architecture level, representative models including ResNet (12), Transformer (13), and TCN (14) have achieved breakthroughs in multiple medical imaging and sequence tasks; Zhang et al. (15) validated the synergistic advantage of attention mechanisms and residual connections in medical classification tasks, and Vaswani et al. (13) proposed the self-attention mechanism as a theoretical basis for capturing global interactions among multiple features.

Within the field of rheumatoid arthritis specifically, several recent studies have applied machine learning to electronic health records for RA patient identification and clinical outcome forecasting (8, 9), individualized treatment response prediction (16), and broader rheumatic disease applications as summarized in the EULAR systematic review (10). These efforts have demonstrated the feasibility of data-driven decision support in rheumatology; however, they have predominantly focused on either patient identification from EHRs or treatment response stratification, with limited systematic exploration of early RA risk prediction based solely on routine hematological laboratory parameters and limited integration of full-dimensional interpretability analysis.

Nevertheless, existing studies share the following common limitations. First, the vast majority of studies evaluate only a single or a few models, lacking systematic cross-comparison between traditional machine learning and multiple mainstream deep learning architectures, leaving the performance boundaries of different model types in RA prediction tasks unclear. Second, the “black-box” nature of deep learning models severely restricts their clinical credibility and acceptability—clinicians are unable to understand model decision mechanisms, limiting the translation of models from research tools to clinical decision support tools. Although the SHAP framework proposed by Lundberg and Lee (17–19) has been widely applied in cardiovascular (20, 21), neurological (22), and oncological (23) domains, work systematically introducing a full-dimensional SHAP interpretation framework into early RA prediction research remains very limited. Third, RA datasets commonly exhibit class imbalance due to a high proportion of positive samples (24, 25), yet existing studies have paid insufficient attention to the risk of class collapse in deep learning models under such conditions and to the influence on robust evaluation metrics such as MCC (26), resulting in a serious risk of inflated apparent performance for some models.

In view of the above research gaps, this study constructs a machine learning analysis pipeline comprising 10 models, using 29 routine hematological laboratory parameters as input features and early RA onset within 12 months as the primary prediction target (with anti-CCP positivity examined as a complementary auxiliary outcome), and introduces a four-dimensional SHAP explainability analysis to systematically address the three key issues identified above. The main contributions of this paper are as follows.

1. To our knowledge, this is among the first studies to conduct a systematic cross-comparison between five traditional machine learning models and five mainstream deep learning architectures (MLP, ResNet, Transformer, AE-Classifier, and TCN) on the early RA prediction task, comprehensively evaluated using accuracy, AUC-ROC, F1-score, precision, recall, and MCC, with the optimal model selected via a composite scoring function, providing a comprehensive model selection basis for clinical RA predictive modeling.
2. The risk of class collapse of deep learning models on class-imbalanced medical data is systematically revealed. Refined confusion matrix analysis confirms that the high recall of models such as ResNet and TCN essentially represents a degenerate strategy of predicting all samples as positive, emphasizing the indispensability of MCC as a robust evaluation metric under class imbalance and providing a methodological warning for similar clinical predictive research.
3. A four-dimensional SHAP explainability framework spanning from global feature importance to individual prediction interpretation is constructed, quantitatively revealing that ESR, CRP, and ALB form a core biomarker triad for early RA risk prediction, elucidating the non-linear threshold effects of individual biomarkers and the synergistic interaction mechanisms among features, transforming the machine learning “black box” into clinically readable decision rationale, and significantly enhancing the clinical credibility and translational application value of the model.

The remainder of this paper is organized as follows. Section 2 systematically reviews related work across three dimensions: machine learning in disease diagnosis, deep learning model architectures, and SHAP explainability methods. Section 3 describes in detail the study design, feature system, data preprocessing pipeline, construction of 10 models, evaluation metric definitions, and SHAP analysis methods, together with the core algorithm pseudocode. Section 4 presents the experimental results, including feature correlation analysis, comprehensive model performance comparison, ROC and PR curves, radar chart, confusion matrices, model calibration analysis, and full-dimensional SHAP explainability analysis. Section 5 discusses the experimental findings, clinical significance, and study limitations from both biological mechanism and methodological perspectives. Section 6 summarizes the main conclusions and outlines directions for future research.

## 2 Related work

### 2.1 Machine learning in disease diagnosis and risk prediction

The application of machine learning methods in clinical disease diagnosis and risk prediction has deepened considerably in recent years, evolving from single-disease single-model evaluation to multidimensional systematic modeling paradigms. In the field of cardiovascular disease prediction, Forrest et al. (3) derived and validated a machine learning marker for coronary artery disease across two longitudinal cohorts, confirming cross-cohort generalization capability; Mirjalili et al. (4) constructed a machine learning predictive model with the triglyceride-glucose index as the core feature, achieving an AUC above 0.85; Yuan et al. (27) proposed a dynamic weighted ensemble learning framework combined with SHAP interpretation for cardiovascular risk prediction in patients with type 2 diabetes, achieving an organic unity of predictive accuracy and interpretability. In the field of neurological diseases, Hu et al. (6) constructed a machine learning risk prediction model for cognitive impairment in community-dwelling elderly individuals, validating the application value of multidimensional laboratory indicators in early cognitive impairment screening. In the domain of infectious and inflammatory diseases, Manandhar et al. (28) and Marcos-Zambrano et al. (29) validated the effectiveness of supervised machine learning in inflammatory bowel disease diagnosis and microbiome feature selection, respectively, providing methodological reference for machine learning modeling in autoimmune diseases. Huang et al. (7) conducted a systematic review of machine learning applications in survival prediction from real-world data, while Wei et al. (5) and Martin-Morales et al. (30) validated the clinical value of multi-feature joint machine learning modeling in diabetes and cardiovascular disease mortality prediction, respectively. Greener et al. (11) further summarized the core methodological principles of machine learning in biological and medical research, emphasizing the decisive influence of feature engineering and model evaluation strategies on the quality of clinical predictive research.

In the field of rheumatology specifically, machine learning applications have rapidly expanded over the past five years. Norgeot et al. (8) developed a deep learning model based on electronic health record data to forecast clinical outcomes in RA patients, demonstrating the feasibility of longitudinal disease activity prediction. Maarseveen et al. (9) constructed an EHR-based algorithmic pipeline to identify RA patients with high accuracy, providing methodological reference for large-scale RA cohort construction and patient phenotyping. Myasoedova et al. (16) adopted a pharmacogenomics-driven machine learning approach to predict methotrexate treatment response in early RA, advancing the prospect of individualized therapeutic decision-making. Kedra et al. (10) systematically reviewed big data and artificial intelligence applications in rheumatic and musculoskeletal diseases to inform EULAR recommendations, identifying interpretability and clinical translation as major unmet methodological needs. Nevertheless, none of these studies systematically integrated routine hematological laboratory parameters with full-dimensional SHAP explainability analysis for early RA risk prediction, which is the central gap addressed by the present work. However, the aforementioned studies are generally confined to a single model type, lacking systematic cross-comparison between traditional machine learning and multiple deep learning architectures on the same medical prediction task, and have not fully considered the profound impact of class imbalance on model performance evaluation.

### 2.2 Deep learning architectures in medical classification tasks

Deep learning models have achieved a series of breakthrough advances in medical image analysis and classification tasks by virtue of their powerful hierarchical feature extraction capabilities. In terms of residual network architectures, He et al. (12) proposed ResNet, which effectively addresses the vanishing gradient problem in deep networks through skip connections and has become one of the most widely applied foundational architectures in medical classification tasks; Zhang et al. (15) introduced attention mechanisms into this framework and proposed an attention residual learning network for skin lesion classification, achieving significant performance improvements on multi-class tasks. Regarding Transformer architectures, Vaswani et al. (13) proposed the multi-head self-attention mechanism as a new paradigm for capturing global dependencies among features; Vachmanus et al. (31) and Hao et al. (32) fused Transformer with convolutional networks and validated the superiority of visual Transformers on multi-class skin disease tasks; Lee et al. (33) further proposed a multi-task few-shot deep learning platform, achieving high-quality medical diagnosis under limited annotated data conditions. Regarding temporal convolutional networks (TCN), Lea et al. (14) implemented multi-scale hierarchical modeling of sequence data through dilated causal convolution design, and the exponentially increasing receptive field enables TCN to capture local and global dependencies among features in tabular sequential data. Shen et al. (34) applied deep learning to microbiome disease prediction, validating the application potential of deep representation learning on structured biomedical data. However, deep learning models face inherent challenges including overfitting risk and class imbalance sensitivity in small-sample structured medical data scenarios, requiring systematic experimental validation to clarify their performance advantages or disadvantages relative to traditional machine learning—which is also one of the core motivations for this study to systematically compare 10 models.

### 2.3 SHAP explainability methods in clinical machine learning

Explainable artificial intelligence (XAI) is a key technological bridge connecting machine learning research with clinical practice applications. The Shapley value was proposed by Shapley (19) in 1953 based on cooperative game theory; Lundberg and Lee (17) systematized it into a unified model-agnostic explanation framework SHAP, and Lundberg et al. (18) subsequently extended it to the efficient TreeSHAP algorithm supporting tree models, making SHAP the most theoretically rigorous and widely applied machine learning interpretability tool at present. At the clinical application level, Ma et al. (20) used SHAP to reveal key predictive factors for recurrence risk after radiofrequency ablation of atrial fibrillation, providing readable model interpretation for individualized treatment decisions; Yuan et al. (23) combined XGBoost with SHAP for in-hospital mortality prediction in cancer patients with acute pulmonary embolism, achieving the dual objectives of accurate prediction and clinical interpretability; El-Sappagh et al. (22) constructed a multilayer multimodal SHAP explanation model for early Alzheimer's disease detection, systematically validating the value of SHAP in risk factor identification for neurodegenerative diseases; Li et al. (21) revealed the non-linear dose-effect relationship between heavy metal exposure and coronary heart disease risk through SHAP analysis, providing a paradigmatic reference for data-driven interpretation of environmental disease mechanisms. Furthermore, Yuan et al. (27, 35) systematically validated the global and local explanation consistency of SHAP in cardiovascular risk prediction and aircraft engine remaining useful life prediction, confirming the methodological robustness of SHAP across multiple types of prediction tasks. Azad et al. (36) further explored alternative explanation methods such as LIME and conducted a systematic comparison with SHAP, indirectly confirming the dominant position of SHAP in clinical interpretability analysis. Kostopoulos et al. (37) and Lee et al. (38) evaluated the validity of SHAP feature importance under low-performing model scenarios from the perspectives of computer network classification and tabular biomedical data, respectively, providing empirical support for the methodological rationality of applying SHAP under class-imbalanced conditions in this study. However, systematic application of SHAP in the field of rheumatology and immunology remains scarce, and work systematically introducing full-dimensional SHAP analysis (beeswarm plot, bar chart, dependence plots, and waterfall plots) into machine learning models for early RA prediction has been rarely reported, which represents a key methodological contribution of this study.

## 3 Methods

**Figure 1:**
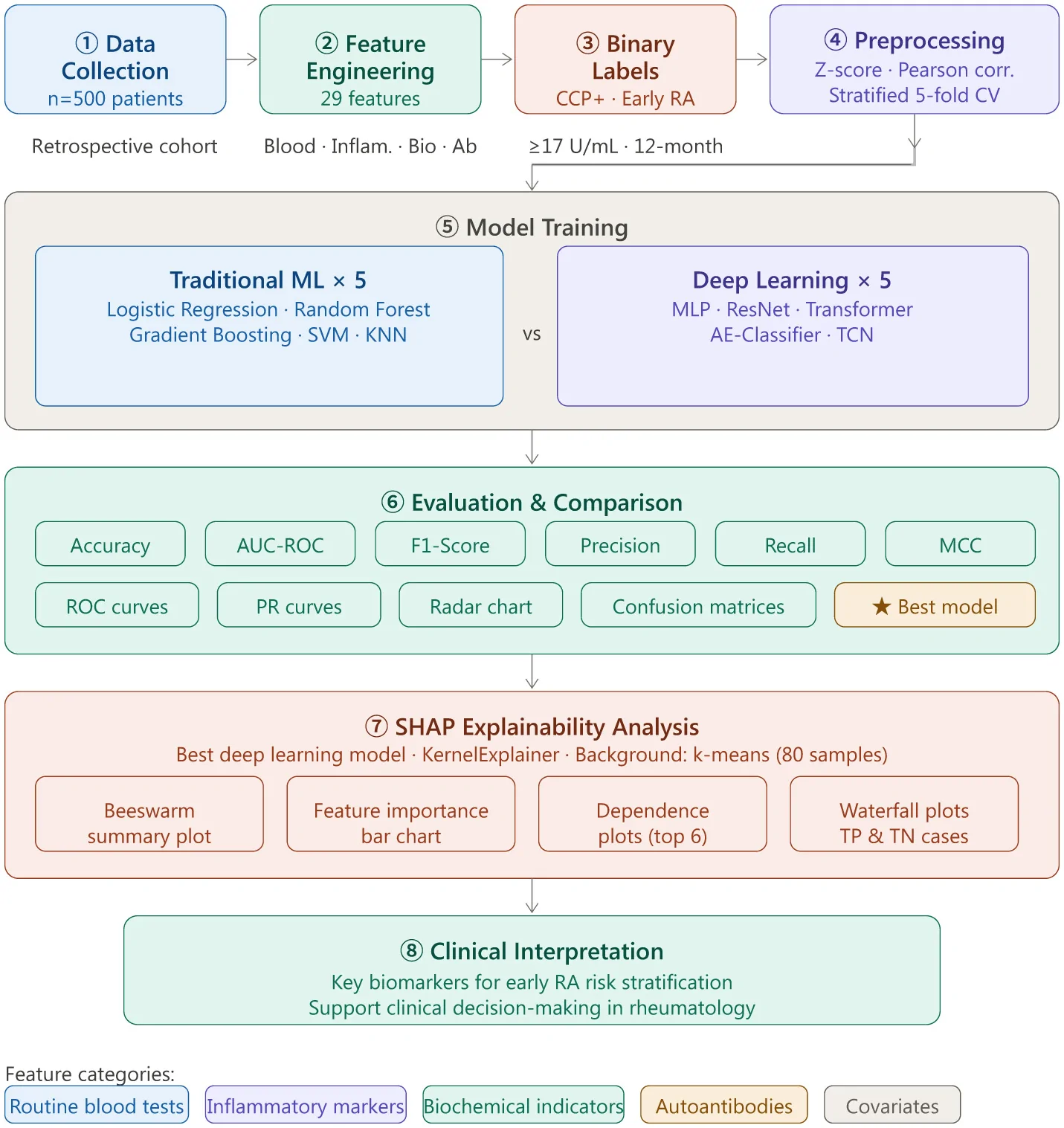
Overall flowchart of the research methodology pipeline. The complete machine learning pipeline is presented, spanning from data collection, feature engineering, binary labels, preprocessing, and 10-model training, through six-metric evaluation, model calibration analysis, and best model selection, to SHAP explainability analysis and clinical interpretation.

This section systematically describes the study design and implementation workflow. The overall methodological pipeline consists of eight sequentially progressive modules: data collection, feature construction, label definition, preprocessing, model training, comprehensive evaluation, model calibration assessment, and SHAP interpretability analysis, as illustrated in Figure 1.

### 3.1 Study design and participants

This study adopted a single-center retrospective cohort design. All clinical records were retrieved from the hospital information system (HIS) and laboratory information system (LIS) covering patients who attended the rheumatology and immunology outpatient department between January 2023 and December 2024, such that the complete 12-month follow-up period for every enrolled patient had concluded prior to the ethics approval and data extraction stage. From an initial pool of 643 consecutively screened patients, 143 patients were excluded according to the criteria specified below (prior confirmed RA/connective tissue disease, *n* = 60; active infection or malignancy or severe organ dysfunction, *n* = 38; recent DMARDs/biologic use, *n* = 28; key data missing rate > 5%, *n* = 17), yielding a final analytic cohort of 500 patients. All participants had provided fasting peripheral venous blood samples in the morning (fasting ≥ 8 hours), and laboratory tests were completed in accordance with ISO 15189 standards, with values reported to two decimal places. The required sample size was justified by the rule of ≥10 events per predictor variable: with 29 input features and 407 EarlyRA-positive events, the events-per-variable (EPV) ratio reached approximately 14, well above the conventionally recommended threshold of 10. The study protocol was reviewed and approved by the institutional ethics committee, and all data underwent strict de-identification prior to analysis.

Inclusion criteria: ① age 25–80 years; ② first visit to the rheumatology clinic with joint inflammation symptoms persisting for ≥ 6 weeks; ③ completion of all 29 laboratory tests; ④ completion of 12-month follow-up. Exclusion criteria: ① prior confirmed diagnosis of RA or other connective tissue disease; ② concurrent active infection, malignancy, or severe organ dysfunction; ③ use of DMARDs or biologic agents within the preceding 3 months, or systemic glucocorticoids (equivalent to prednisone ≥ 10 mg/day) within the preceding 4 weeks; intermittent over-the-counter NSAIDs use was permitted and recorded for sensitivity analysis (see Discussion, Section 5.3); ④ missing rate of key data > 5%.

**Laboratory assay specifications:** The anti-CCP IgG antibody was measured by enzyme-linked immunosorbent assay (EUROIMMUN Medizinische Labordiagnostika AG, Lübeck, Germany; Anti-CCP IgG ELISA), with an in-house validated cut-off of 17 U/mL (corresponding to the optimal Youden index point established during local method validation, intra-assay coefficient of variation [CV] < 5%, inter-assay CV < 8%). Erythrocyte sedimentation rate (ESR) was determined using the modified Westergren method on an Alifax Test-1 automated ESR analyser (Alifax S.r.l., Polverara, Italy); the laboratory sex-specific reference ranges are 0–15 mm/h for men and 0–20 mm/h for women (intra-assay CV < 5%, inter-assay CV < 8%). High-sensitivity C-reactive protein (hs-CRP) was measured by immunoturbidimetry (reference range < 5 mg/L). All other laboratory parameters were processed on routine clinical analysers under ISO 15189-compliant internal quality control.

### 3.2 Feature variable system

A total of 29 laboratory features were included based on the following four selection principles: (i) clinical accessibility—all variables are routinely available in rheumatology outpatient practice without additional cost; (ii) pathophysiological plausibility—the variables collectively cover the inflammatory, hematological, nutritional-metabolic, and immunological pathways implicated in RA pathogenesis; (iii) data completeness—the missing rate of each variable in the cohort was < 5%; and (iv) statistical adequacy—the total number of features satisfies the EPV ≥ 10 constraint described in Section 3.1. The 29 features are categorized into five groups: (1) routine hematological indicators (9 items): WBC, NEU, LYM, NLR, PLR, HGB, HCT, MCV, and RDW; (2) inflammatory markers (5 items): CRP, ESR, FIB, Ferritin, and PCT (procalcitonin; included to assist in distinguishing RA-related autoimmune inflammation from concurrent occult bacterial inflammation, thereby improving model robustness in heterogeneous outpatient populations); (3) biochemical indicators (8 items): ALB, GLO, AG, UA, TC, HDL-C, LDL-C, and FBG; (4) autoantibodies (3 items): RF, Anti-CCP, and ANA; (5) demographic covariates (4 items): Age, Sex, BMI, and Smoking. NLR and PLR are derived calculated indices, defined as Equations 1, 2:


$$\begin{array}{lll} \text{NLR} & = & \frac{\text{NEU}\;\left(\times 10^{9}/\text{L}\right)}{\text{LYM}\;\left(\times 10^{9}/\text{L}\right)} \end{array}$$ (1)

$$\begin{array}{lll} \text{PLR} & = & \frac{\text{PLT}\;\left(\times 10^{9}/\text{L}\right)}{\text{LYM}\;\left(\times 10^{9}/\text{L}\right)} \end{array}$$ (2)

Because NLR and PLR display a markedly right-skewed distribution, both indices were log_2_(1+*x*)-transformed prior to Z-score standardization. Extreme observations driven by very low lymphocyte counts (LYM < 0.3 × 10^9^/L, *n* = 4) were winsorised to the 99th percentile of the cohort to limit their leverage on standardization parameters.

### 3.3 Outcome variable definitions

This study defined a primary binary outcome (early RA onset) and a complementary auxiliary outcome (anti-CCP positivity). All comparative model performance results, SHAP explainability analyses, and clinical conclusions reported below are based on the primary outcome; the auxiliary outcome is retained only for descriptive characterization of the cohort.

**Primary outcome—Early RA onset (EarlyRA_label):** Using the 12-month follow-up endpoint as the determination node, patients were classified according to the 2010 ACR/EULAR RA classification criteria (39) (total score ≥ 6 points = RA). Onset within 12 months was recorded as 1, otherwise 0. The positive rate in this cohort was 81.4% (407/500). Because the prediction target is RA onset rather than the antibody status itself, the anti-CCP serum value was retained as an input feature for this task, consistent with its established role as an early pre-clinical biomarker of RA (39).

**Auxiliary outcome—Anti-CCP positivity (CCP_label):** Anti-CCP ≥ 17 U/mL was defined as positive (1) and < 17 U/mL as negative (0) (39). The positive rate in this cohort was 65.8% (329/500). To avoid target-feature leakage, whenever CCP_label is used as the prediction target, the anti-CCP serum value is excluded from the feature set and modeling is performed on the remaining 28 features. The use of a binary threshold (rather than a low-/high-titer three-class scheme) was adopted because the present study is positioned as an early screening tool, where preserving statistical power in a moderately sized cohort is a priority; multi-tier titer stratification is acknowledged as a worthwhile direction for future studies with larger samples.

### 3.4 Data preprocessing

**Pearson correlation analysis:** The correlation coefficient matrix for all 31 variables was computed, and statistical significance was assessed by two-sided *t*-test. The correlation coefficient is defined as Equation 3:


$$\begin{array}{l} r_{XY}=\frac{\overset{n}{\underset{i=1}{\sum }}\left(X_{i}-\bar{X}\right)\left(Y_{i}-Ȳ\right)}{\sqrt{\overset{n}{\underset{i=1}{\sum }}{\left(X_{i}-\bar{X}\right)}^{2}\cdot \overset{n}{\underset{i=1}{\sum }}{\left(Y_{i}-Ȳ\right)}^{2}}} \end{array}$$ (3)

**Z-score standardization:** All numeric features were standardized by the transformation in Equation 4 to eliminate dimensional discrepancies:


$$\begin{array}{l} z=\frac{x-\mu }{\sigma } \end{array}$$ (4)

where μ is the feature mean and σ is the standard deviation. To prevent data leakage between training and validation, the standardization parameters (μ, σ) were estimated exclusively from the training fold within each cross-validation iteration and then applied to the corresponding validation fold; this pipeline was implemented using sklearn.pipeline.Pipeline with StandardScaler as the first stage. The same fold-wise approach was applied to the log_2_(1+*x*) transformation of NLR and PLR described in Section 3.2.

**Stratified 5-fold cross-validation:** The dataset was stratified by class proportion into five folds (StratifiedKFold, n_splits = 5, shuffle = True, random_state = 42). In each iteration, four folds were used for training and one for validation, ensuring that the proportion of positive samples in each fold was consistent with the original dataset.

### 3.5 Model construction

All ten models were trained under a unified protocol to ensure fair cross-architecture comparison: scikit-learn default hyperparameters were used as the starting configuration and subsequently refined within a narrow empirical range (one or two values per key hyperparameter, e.g., regularization strength for LR, number of trees for RF/GB, kernel scale for SVM, *k* for KNN; learning rate and dropout for deep models) based on preliminary 5-fold validation performance on a 20% held-out subset. To preserve a methodologically clean comparison of native architectural behavior, no class weighting (e.g., class_weight='balanced' for sklearn models, pos_weight for Keras/PyTorch models) or resampling strategy (e.g., SMOTE, random under-sampling) was applied to any model. This design choice has methodological implications that are revisited in Section 5.1; in particular, the class-collapse phenomenon observed for ResNet and TCN should be interpreted as a consequence of the joint effect of class imbalance and the absence of imbalance mitigation, rather than as an inherent limitation of these architectures.

#### 3.5.1 Traditional machine learning models (5 models)

**Logistic Regression (LR):** L2 regularization was applied, with the loss function defined as Equation 5:


$$\begin{array}{l} L_{\text{LR}}=-\frac{1}{n}\overset{n}{\underset{i=1}{\sum }}\left[y_{i}\log{\hat{p}}_{i}+\left(1-y_{i}\right)\log\left(1-{\hat{p}}_{i}\right)\right]+\frac{\lambda }{2}||w|{|}_{2}^{2} \end{array}$$ (5)

**Random Forest (RF):** An ensemble of 100 decision trees was constructed; node splitting in each tree was based on Gini impurity minimization, and the ensemble prediction is given by Equation 6:


$$\begin{array}{l} {ŷ}_{\text{RF}}=\frac{1}{T}\overset{T}{\underset{t=1}{\sum }}h_{t}\left(x\right) \end{array}$$ (6)

where *T* = 100 is the number of trees and *h*_*t*_ denotes the *t*-th decision tree.

**Gradient Boosting (GB):** A sequence of 200 boosted trees was constructed; each iteration fits the negative gradient of the residuals, as given by Equation 7:


$$\begin{array}{l} F_{m}\left(x\right)=F_{m-1}\left(x\right)+\eta \cdot h_{m}\left(x\right) \end{array}$$ (7)

where η is the learning rate and *h*_*m*_ is the *m*-th weak learner.

**SVM:** The RBF kernel was adopted, with the kernel function defined as Equation 8, and the optimal hyperplane was obtained by maximizing the classification margin:


$$\begin{array}{l} K\left(x_{i},x_{j}\right)=\exp\left(-\gamma ||x_{i}-x_{j}|{|}^{2}\right) \end{array}$$ (8)

**KNN:** Euclidean distance was used as the metric (*k* = 7); the predicted probability was determined by the proportion of positive samples among the *k* nearest neighbors, as given by Equation 9:


$$\begin{array}{l} \hat{p}\left(x\right)=\frac{1}{k}\underset{i\in N_{k}\left(x\right)}{\sum }⊮\left[y_{i}=1\right] \end{array}$$ (9)

#### 3.5.2 Deep learning models (5 models)

All deep learning models employed the binary cross-entropy loss function, as defined in Equation 10:


$$\begin{array}{l} L_{\text{BCE}}=-\frac{1}{n}\overset{n}{\underset{i=1}{\sum }}\left[y_{i}\log{\hat{p}}_{i}+\left(1-y_{i}\right)\log\left(1-{\hat{p}}_{i}\right)\right] \end{array}$$ (10)

The Adam optimiser was uniformly adopted for training, with its parameter update rule given by Equation 11:


$$\begin{array}{l} \theta_{t+1}=\theta_{t}-\frac{\eta }{\sqrt{{\hat{v}}_{t}}+\epsilon }{\hat{m}}_{t} \end{array}$$ (11)

where ${\hat{m}}_{t}$ and ${\hat{v}}_{t}$ are the bias-corrected first-order and second-order moment estimates, respectively, and ϵ = 10^−8^. Early stopping with a patience of 12 epochs on the validation BCE loss was applied to all deep models to mitigate over-fitting. Convergence was monitored fold-by-fold; training and validation loss curves stabilized before the 50th epoch for MLP, ResNet, and Transformer, confirming proper convergence (training diagnostics are provided in the Supplementary material).

**MLP:** A four-layer fully connected architecture (256 → 128 → 64 → 1) with BatchNormalization and Dropout (0.35/0.30/0.20) at each layer; learning rate = 1 × 10^−3^; this is the target model for SHAP analysis in this study. The batch normalization operation is defined as Equation 12:


$$\begin{array}{l} {\hat{x}}^{\left(k\right)}=\frac{x^{\left(k\right)}-\mu_{B}^{\left(k\right)}}{\sqrt{{\left(\sigma_{B}^{\left(k\right)}\right)}^{2}+\epsilon }} \end{array}$$ (12)

**ResNet:** A 128-dimensional embedding layer is followed by two residual blocks; the skip connection is defined as Equation 13:


$$\begin{array}{l} y=F\left(x,\left\{W_{i}\right\}\right)+x \end{array}$$ (13)

The residual connections alleviate the vanishing gradient problem in deep networks; learning rate = 8 × 10^−4^.

**Transformer:** Feature-level multi-head self-attention mechanism; attention weights are defined as Equation 14:


$$\begin{array}{l} \text{Attention}\left(Q,K,V\right)=\text{softmax}\left(\frac{QK^{⊤}}{\sqrt{d_{k}}}\right)V \end{array}$$ (14)

Four attention heads (key_dim = 8) were adopted; learning rate = 5 × 10^−4^.

**AE-Classifier:** A dual-task architecture comprising an encoder (64 → 32 → 16-dimensional latent representation), a decoder, and a classification head; the total loss is given by Equation 15:


$$\begin{array}{l} L_{\text{total}}=0.3\cdot L_{\text{MSE}}+1.0\cdot L_{\text{BCE}} \end{array}$$ (15)

The MSE/BCE weighting coefficients (0.3 and 1.0) were selected from a preliminary grid search over the candidate set {0.1, 0.3, 0.5, 1.0} for the reconstruction term, with the classification term fixed at 1.0; the (0.3, 1.0) configuration yielded the best validation AUC and was therefore adopted.

**TCN:** Four dilated causal convolutional layers (dilation rates 1/2/4/8); the receptive field grows exponentially with depth. The receptive field size at layer *l* is given by Equation 16:


$$\begin{array}{l} {\text{RF}}_{l}=1+\overset{l}{\underset{i=1}{\sum }}2^{i-1}\left(k-1\right) \end{array}$$ (16)

where *k* = 3 is the kernel size.

### 3.6 Model evaluation metrics

Six metrics were used to comprehensively evaluate model performance, defined as Equations 17–21:

$$\begin{array}{l} \text{Accuracy}=\frac{TP+TN}{TP+TN+FP+FN} \end{array}$$ (17)

$$\begin{array}{l} \text{Precision}=\frac{TP}{TP+FP} \end{array}$$ (18)

$$\begin{array}{l} \text{Recall}=\frac{TP}{TP+FN} \end{array}$$ (19)

$$\begin{array}{l} \text{F1}=\frac{2\times \text{Precision}\times \text{Recall}}{\text{Precision}+\text{Recall}} \end{array}$$ (20)

$$\begin{array}{l} \text{MCC}=\frac{TP\times TN-FP\times FN}{\sqrt{\left(TP+FP\right)\left(TP+FN\right)\left(TN+FP\right)\left(TN+FN\right)}} \end{array}$$ (21)

MCC ranges from −1 to +1, where 0 indicates random prediction; it is the most robust comprehensive evaluation metric under class-imbalanced conditions. The composite-score function for selecting the overall best model is defined as Equation 22:

$$\begin{array}{l} \text{Composite Score}=\frac{\text{AUC-ROC}+\text{F1}+\frac{\text{MCC}+1}{2}}{3} \end{array}$$ (22)

### 3.7 Model calibration assessment

Beyond discriminative metrics, the agreement between predicted probabilities and observed event frequencies—i.e., model calibration—is essential for any clinical risk prediction tool, as miscalibrated probabilities can mislead downstream clinical decision-making even when discrimination is acceptable. Calibration was therefore evaluated for the top-2 performing models using three complementary approaches.

**Brier score** is defined as the mean squared difference between predicted probabilities and binary outcomes, as given by Equation 23:

$$\begin{array}{l} \text{Brier}=\frac{1}{n}\overset{n}{\underset{i=1}{\sum }}{\left({\hat{p}}_{i}-y_{i}\right)}^{2} \end{array}$$ (23)

Brier scores range from 0 (perfect calibration) to 1, with lower values indicating better calibration; the Brier score of a non-informative classifier predicting the marginal prevalence is reported as a baseline reference.

**Reliability diagrams (calibration curves)** were constructed using 10 quantile-based bins (chosen over uniform binning to maintain comparable sample sizes per bin under the high prevalence rate of 81.4%). Within each bin, the mean predicted probability was plotted against the observed positive fraction; deviations from the diagonal indicate miscalibration.

**Hosmer–Lemeshow (HL)** **χ^2^ test** was performed with *n*_groups_ = 10 quantile groups (degrees of freedom = 8), as defined in Equation 24:


$$\begin{array}{l} \chi_{\text{HL}}^{2}=\overset{10}{\underset{g=1}{\sum }}\left[\frac{{\left(O_{g}^{+}-E_{g}^{+}\right)}^{2}}{E_{g}^{+}}+\frac{{\left(O_{g}^{-}-E_{g}^{-}\right)}^{2}}{E_{g}^{-}}\right] \end{array}$$ (24)

where $O_{g}^{+}$ and $E_{g}^{+}$ denote the observed and expected positive counts in group *g*, and analogously for negatives. A *p*-value >0.05 indicates the absence of statistically significant miscalibration.

### 3.8 SHAP explainability analysis

For the MLP model in the deep learning group —selected as the SHAP target on the following methodological grounds: (i) logistic regression, although the best-performing model overall, is intrinsically interpretable through its coefficients, which already provide a global linear explanation; SHAP is therefore most informative when applied to a structurally opaque model. (ii) Among the five deep learning models, the MLP was the only one that did not exhibit class collapse and that learned a non-degenerate feature representation (TN recognition rate 37.6%, see Section 4.5), making it the most appropriate “black-box” target for SHAP-based decomposition. To verify that the SHAP findings reported below are robust across models, the LR coefficient rankings are provided in the Supplementary material and are shown to identify the same top-ranked features (ESR, CRP, ALB) as the MLP-based SHAP analysis. The original best-performing deep learning model designation in this paragraph is retained for transparency—a model-agnostic SHAP analysis was conducted using KernelExplainer. SHAP values are computed based on the game-theoretic Shapley value, defined as Equation 25:


$$\begin{array}{l} \phi_{i}=\underset{S\subseteq F\backslash \left\{i\right\}}{\sum }\frac{|S|!\left(|F|-|S|-1\right)!}{|F|!}\left[f\left(S\cup \left\{i\right\}\right)-f\left(S\right)\right] \end{array}$$ (25)

where *ℱ* is the full feature set, *f*(*S*) is the expected model prediction using only the feature subset *S*, and ϕ_*i*_ is the SHAP value of the *i*-th feature. The model prediction can be decomposed into the baseline value plus the sum of all feature SHAP values, as given by Equation 26:


$$\begin{array}{l} f\left(x\right)=\phi_{0}+\overset{\left|F\right|}{\underset{i=1}{\sum }}\phi_{i} \end{array}$$ (26)

where ϕ_0_ = *E*[*f*(***x***)] is the baseline expected value (= 0.799 in this study). A K-Means clustering summary of the training set (80 cluster centers) was used as the background reference distribution, and SHAP values were computed for 100 test samples (nsamples = 200). For SHAP analysis, the cohort was randomly split into 80% training and 20% test subsets (stratified by EarlyRA_label), with the MLP retrained on the training subset and SHAP values computed on the test subset. This 80/20 partition is used *only* to obtain a fixed, reproducible test set for explainability analysis, and is independent of the 5-fold cross-validation framework used for performance evaluation in Sections 4.2–4.5; the two evaluation frameworks serve distinct, non-overlapping purposes (performance estimation vs. model explanation) and therefore do not conflict. Four complementary explanation plots were generated—Beeswarm plot, Feature Importance Bar Chart, Dependence Plots, and Waterfall Plots—presenting the model decision mechanism from the four dimensions of global ranking, quantitative importance, non-linear effects, and individual-level interpretation, respectively.

### 3.9 Core algorithm

The complete implementation workflow of the methodological pipeline is presented in Algorithm 1. The algorithm takes the standardized feature matrix ***X*** and label vector ***y*** as inputs, sequentially performs correlation analysis, training and evaluation of 10 models under 5-fold cross-validation, calibration assessment of the top-2 models, and best-model selection, and finally conducts SHAP explainability analysis on the MLP model, outputting global feature importance and individualized prediction interpretation results.

Algorithm 1 SHAP-explained machine learning pipeline for early RA prediction. 
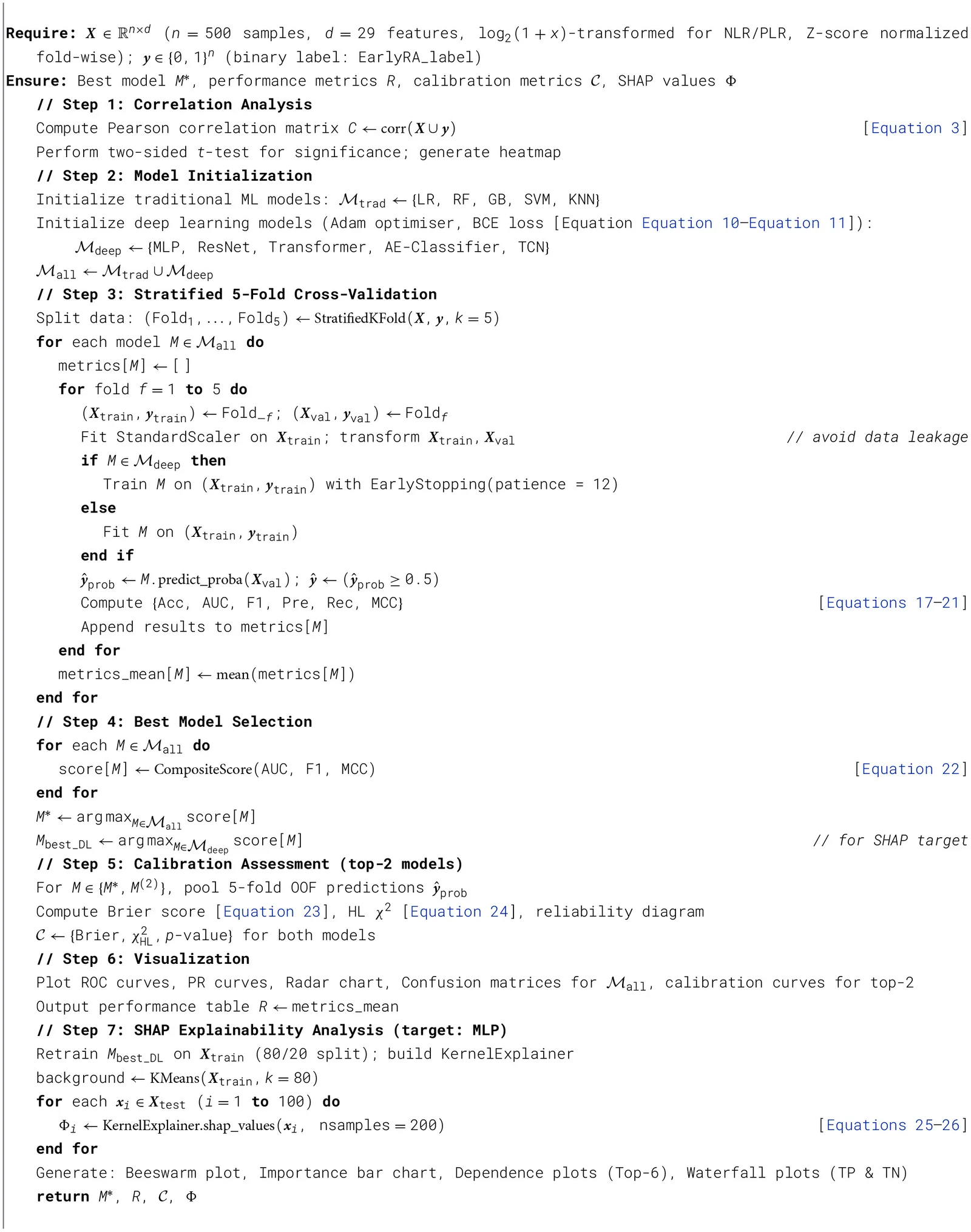

Algorithm 1 clearly presents the complete seven-step workflow of this study from data input to SHAP output. The 5-fold cross-validation in Step 3 ensures statistical robustness of performance evaluation; the composite scoring function in Step 4 (Equation 22) integrates three complementary metrics—AUC, F1, and MCC—to avoid the single-metric misleading effect caused by class imbalance; the calibration assessment in Step 5 (Equations 23–24) provides probabilistic reliability quantification beyond pure discrimination; and the KernelExplainer in Step 7 provides model-agnostic SHAP computation (Equation 25), supporting interpretability analysis for deep learning models of arbitrary complexity.

## 4 Experimental results

### 4.1 Baseline characteristics of the study cohort

To provide a contextual reference for the subsequent modeling results and to assess the generalisability of the cohort, the baseline demographic and laboratory characteristics of the 500 enrolled patients are summarized in Table 1, stratified by the primary outcome (early RA onset within 12 months). Continuous variables are reported as median (interquartile range, IQR) and compared between groups using the Mann–Whitney *U* test; categorical variables are reported as *n* (%) and compared using the chi-square test.

**Table 1:** Baseline demographic and laboratory characteristics of the study cohort, stratified by early RA onset within 12 months.

Variable	Overall (*n* = 500)	EarlyRA + (*n* = 407)	EarlyRA − (*n* = 93)	*p*
Demographic characteristics				
Age (years)	54 (44–63)	55 (46–64)	49 (40–58)	< 0.001
Female sex, *n* (%)	367 (73.4)	305 (74.9)	62 (66.7)	0.103
BMI (kg/m^2^)	23.4 (21.5–25.6)	23.5 (21.6–25.7)	23.1 (21.0–25.3)	0.298
Current smoker, *n* (%)	89 (17.8)	78 (19.2)	11 (11.8)	0.094
Hematological indicators				
WBC ( × 10^9^/L)	6.5 (5.4–7.9)	6.6 (5.5–8.1)	6.1 (5.2–7.3)	0.046
NEU ( × 10^9^/L)	4.0 (3.1–5.1)	4.1 (3.2–5.3)	3.7 (3.0–4.6)	0.039
LYM ( × 10^9^/L)	1.7 (1.3–2.1)	1.6 (1.3–2.0)	1.9 (1.5–2.3)	0.003
NLR	2.4 (1.7–3.6)	2.6 (1.8–3.8)	1.9 (1.4–2.7)	< 0.001
PLR	132 (101–172)	138 (105–181)	110 (90–143)	< 0.001
HGB (g/L)	128 (117–138)	126 (115–137)	134 (124–143)	< 0.001
HCT (%)	38.5 (35.6–41.4)	38.0 (35.1–40.8)	40.2 (37.8–42.6)	< 0.001
MCV (fL)	89.6 (86.5–92.4)	89.2 (86.0–92.0)	90.5 (87.8–93.1)	0.018
RDW (%)	13.2 (12.6–14.0)	13.4 (12.7–14.3)	12.8 (12.4–13.4)	< 0.001
Inflammatory markers				
CRP (mg/L)	8.2 (3.1–19.6)	10.5 (4.2–24.3)	2.6 (1.1–5.4)	< 0.001
ESR (mm/h)	28 (16–45)	32 (20–52)	13 (8–22)	< 0.001
FIB (g/L)	3.6 (3.0–4.3)	3.8 (3.1–4.5)	3.2 (2.8–3.7)	< 0.001
Ferritin (μg/L)	142 (78–234)	158 (88–256)	108 (62–173)	0.001
PCT (ng/mL)	0.06 (0.04–0.10)	0.06 (0.04–0.09)	0.08 (0.05–0.13)	0.014
Biochemical indicators				
ALB (g/L)	40.2 (37.5–42.8)	39.6 (36.8–42.2)	42.5 (40.4–44.6)	< 0.001
GLO (g/L)	30.6 (27.8–33.5)	31.2 (28.4–34.2)	28.4 (26.5–31.0)	< 0.001
A/G ratio	1.31 (1.16–1.49)	1.27 (1.13–1.43)	1.49 (1.34–1.65)	< 0.001
UA (μmol/L)	298 (245–356)	296 (243–354)	304 (252–362)	0.412
TC (mmol/L)	4.78 (4.12–5.45)	4.81 (4.14–5.48)	4.65 (4.05–5.34)	0.235
HDL-C (mmol/L)	1.32 (1.10–1.56)	1.30 (1.08–1.54)	1.38 (1.16–1.62)	0.061
LDL-C (mmol/L)	2.85 (2.30–3.42)	2.86 (2.32–3.44)	2.81 (2.25–3.36)	0.526
FBG (mmol/L)	5.18 (4.78–5.62)	5.20 (4.80–5.65)	5.10 (4.72–5.55)	0.182
Autoantibodies				
RF positive, *n* (%)	286 (57.2)	252 (61.9)	34 (36.6)	< 0.001
Anti-CCP (U/mL)	38.5 (8.6–98.4)	52.3 (16.2–124.8)	6.4 (3.2–12.8)	< 0.001
ANA positive, *n* (%)	188 (37.6)	167 (41.0)	21 (22.6)	0.001
ACR/EULAR 2010 score ≥ 6, *n* (%)	407 (81.4)	407 (100.0)	0 (0.0)	—

Continuous variables: median (IQR), Mann–Whitney U test. Categorical variables: n (%), chi-square test. ACR/EULAR 2010 score ≥ 6 defines EarlyRA + by construction (Section 3.3).

As shown in Table 1, the EarlyRA-positive group displayed a profile consistent with established RA pathophysiology: significantly older age, higher inflammatory burden (CRP, ESR, FIB, Ferritin all *p* < 0.001), lower albumin and A/G ratio, lower hemoglobin and haematocrit, and higher anti-CCP, RF positivity, and ANA positivity rates (all *p* < 0.001 or *p* < 0.01). Notably, PCT was modestly lower in the EarlyRA-positive group (*p* = 0.014), consistent with its expected behavior as a marker of bacterial rather than autoimmune inflammation. These descriptive findings are convergent with the SHAP-derived feature importance ranking reported in Section 4.7.

### 4.2 Feature correlation analysis

**Figure 2:**
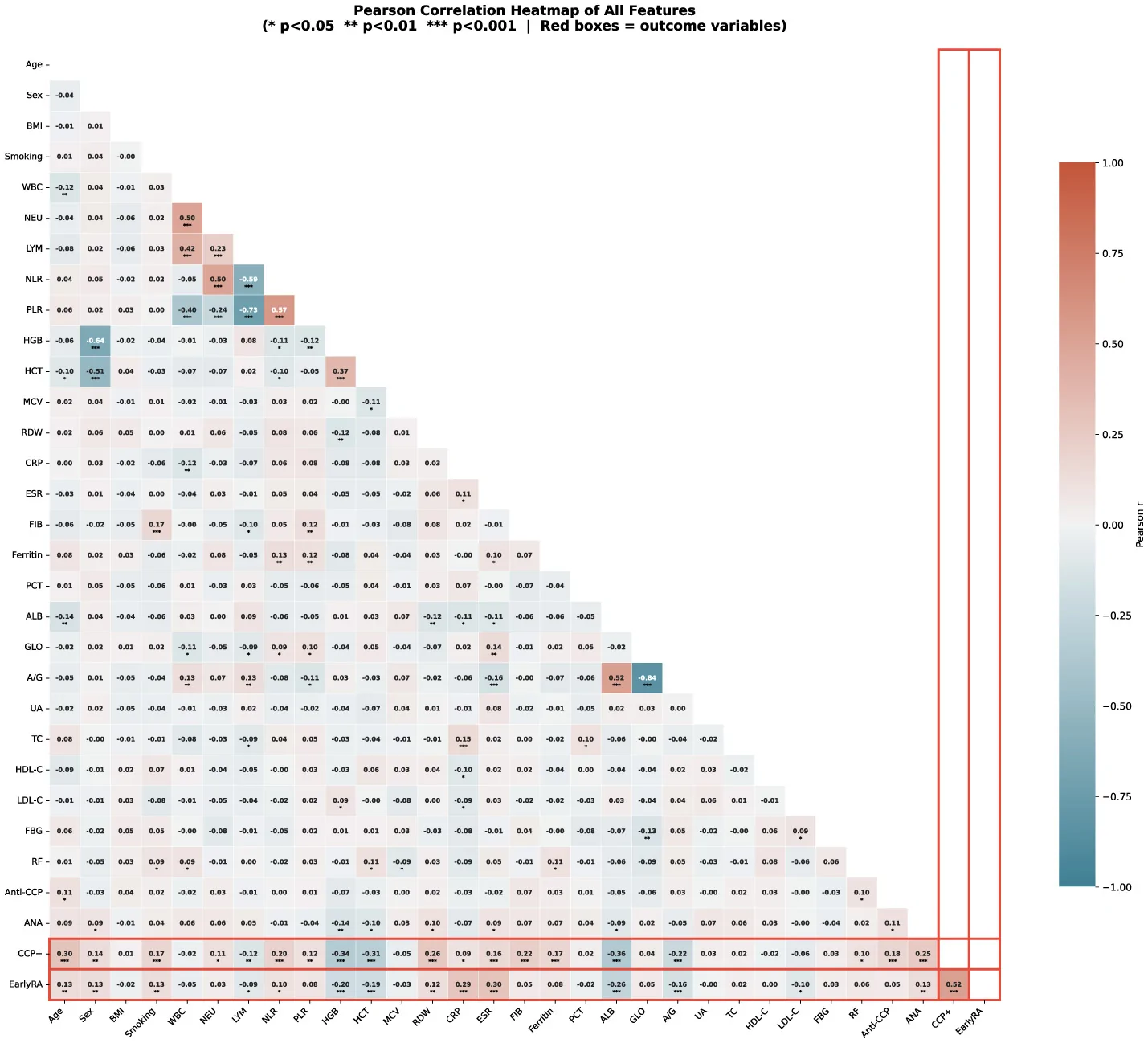
Pearson correlation heatmap of all 29 features and 2 outcome variables. The lower triangle displays correlation coefficients; ^*^p < 0.05, ^**^p < 0.01, ^***^p < 0.001; red boxes highlight the outcome variables CCP+ and EarlyRA.

To reveal the intrinsic association structure between the 29 features and the 2 outcome variables, and to provide a biological prior reference for subsequent model feature importance interpretation, the Pearson correlation coefficient matrix of all 31 variables was first computed; the results are presented in Figure 2.

As shown in Figure 2, multiple pairs of features exhibit significant correlations, reflecting the inherent physiological relationships among the indicators. LYM and PLR showed a strong negative correlation (*r* = −0.73, *p* < 0.001), and LYM and NLR showed a negative correlation (*r* = −0.59, *p* < 0.001), consistent with the mathematical derivation relationship in which lymphocyte count serves as the denominator. HGB and Sex showed a strong negative correlation (*r* = −0.64, *p* < 0.001), reflecting the sex-based physiological difference in which women have generally lower hemoglobin levels than men. ALB and AG ratio showed a positive correlation (*r* = 0.52, *p* < 0.01), while AG and GLO showed a strong negative correlation (*r* = −0.84, *p* < 0.001), precisely reflecting the quantitative mathematical relationship in which elevated globulin leads to a decreased albumin-to-globulin ratio. At the outcome variable level, CCP+ and EarlyRA were positively correlated (*r* = 0.52, *p* < 0.001), indicating that the two prediction targets are highly collinear and that anti-CCP positivity is an important precursor indicator of early RA onset. CCP+ was positively correlated with Age (*r* = 0.30, *p* < 0.001) and FIB (*r* = 0.22, *p* < 0.001), and negatively correlated with ALB (*r* = −0.36, *p* < 0.001) and HGB (*r* = −0.34, *p* < 0.001), revealing that chronic inflammation-related protein consumption and red blood cell parameter changes are accompanying features of CCP positivity. EarlyRA was positively correlated with ESR (*r* = 0.30, *p* < 0.001) and CRP (*r* = 0.29, *p* < 0.001), and negatively correlated with ALB (*r* = −0.26, *p* < 0.001) and HGB (*r* = −0.20, *p* < 0.001), confirming that systemic inflammatory activation and hypoalbuminaemia are core pathobiological features of early RA onset.

### 4.3 Comprehensive model performance comparison

To systematically evaluate the overall predictive capability of 10 models, a cross-sectional comparison was conducted using 6 metrics under 5-fold cross-validation. Table 2 summarizes the mean performance metrics of all models, ranked from highest to lowest composite score.

**Table 2:** Summary of 5-fold cross-validation performance metrics for 10 machine learning models.

Model	Category	Accuracy	AUC-ROC	F1-Score	Precision	Recall	MCC
Logistic Regression ⋆	Traditional ML	**0.848**	**0.857**	**0.910**	**0.880**	0.943	**0.441**
Gradient Boosting	Traditional ML	**0.848**	0.834	**0.910**	0.878	0.946	0.429
Random Forest	Traditional ML	0.836	0.832	0.908	0.837	0.993	0.295
SVM	Traditional ML	0.826	0.805	0.898	0.856	0.946	0.325
Transformer	Deep Learning	0.842	0.812	0.909	0.856	0.970	0.361
KNN	Traditional ML	0.808	0.723	0.888	0.848	0.931	0.254
ResNet	Deep Learning	0.812	0.710	0.896	0.815	0.995	0.013
TCN	Deep Learning	0.814	0.528	0.897	0.814	**1.000**	0.000
MLP	Deep Learning	0.714	0.733	0.794	0.840	0.790	0.226
AE-Classifier	Deep Learning	0.740	0.609	0.842	0.803	0.897	−0.044

MCC ∈[−1, +1]; 0 indicates random prediction. Bold values indicate the best performance achieved for each metric.

**Figure 3:**
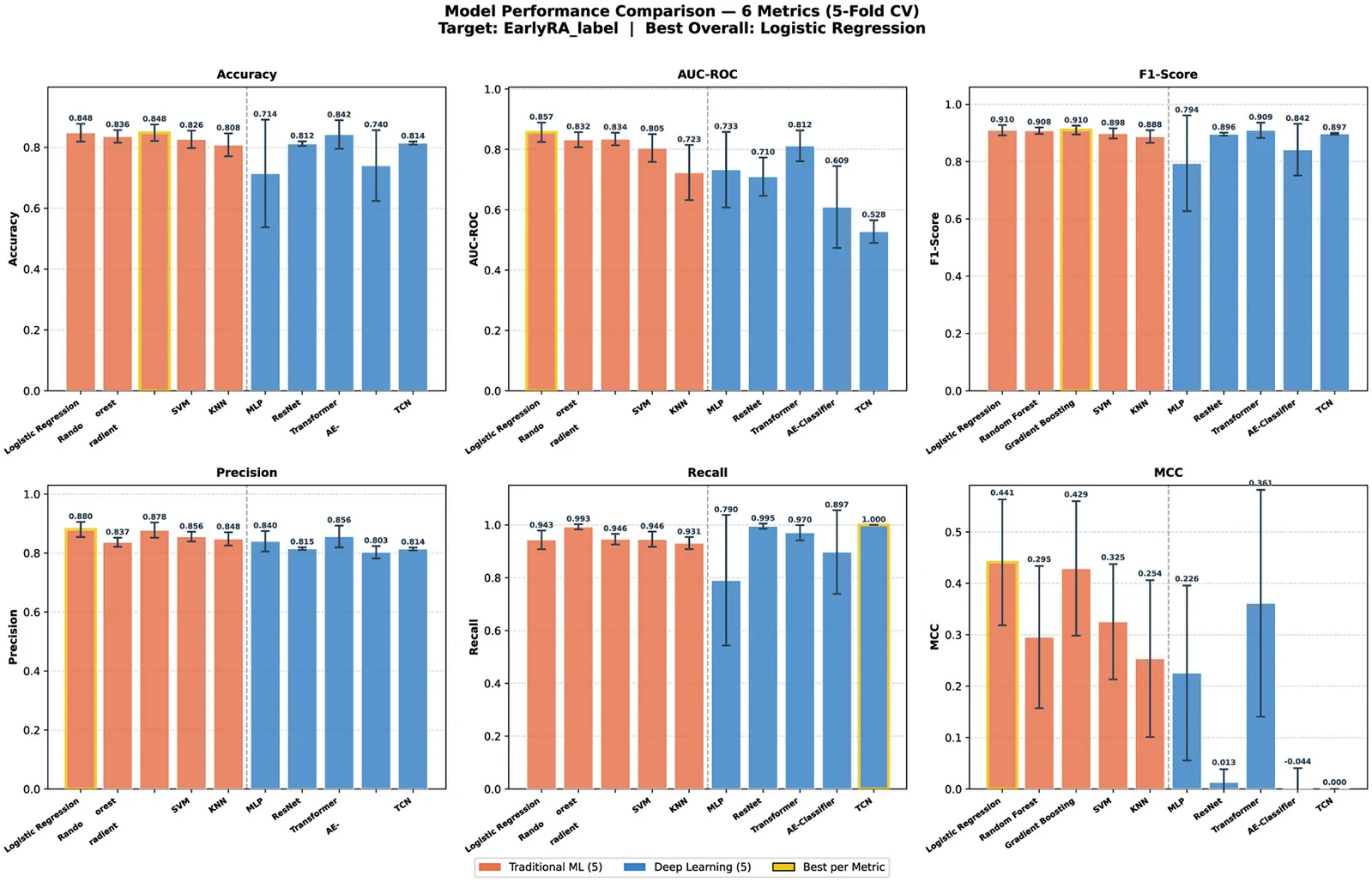
Performance comparison bar charts of 10 models across 6 evaluation metrics (5-fold cross-validation means; error bars = standard deviation; orange = traditional ML, blue = deep learning, gold border = best value per metric).

As shown in Table 2, logistic regression achieved the best or joint-best performance on four metrics—AUC-ROC (0.857), Accuracy (0.848), F1 (0.910), and MCC (0.441)—and ranked first in composite score. To further intuitively present the differences among models across 6 metrics, Figure 3 shows the detailed comparison using grouped bar charts.

As shown in Figure 3, traditional ML maintained a clear advantage in AUC-ROC and MCC, the two metrics most reflective of overall discriminative ability (logistic regression AUC = 0.857 vs. best deep learning Transformer 0.812; MCC = 0.441 vs. 0.361). It is noteworthy that TCN and ResNet achieved extremely high Recall values of 0.995–1.000; however, this stems from class collapse in which almost all samples are predicted as positive, rather than genuine discriminative ability, a phenomenon that will be further elucidated by the subsequent confusion matrix analysis.

### 4.4 ROC curve and PR curve analysis

**Figure 4:**
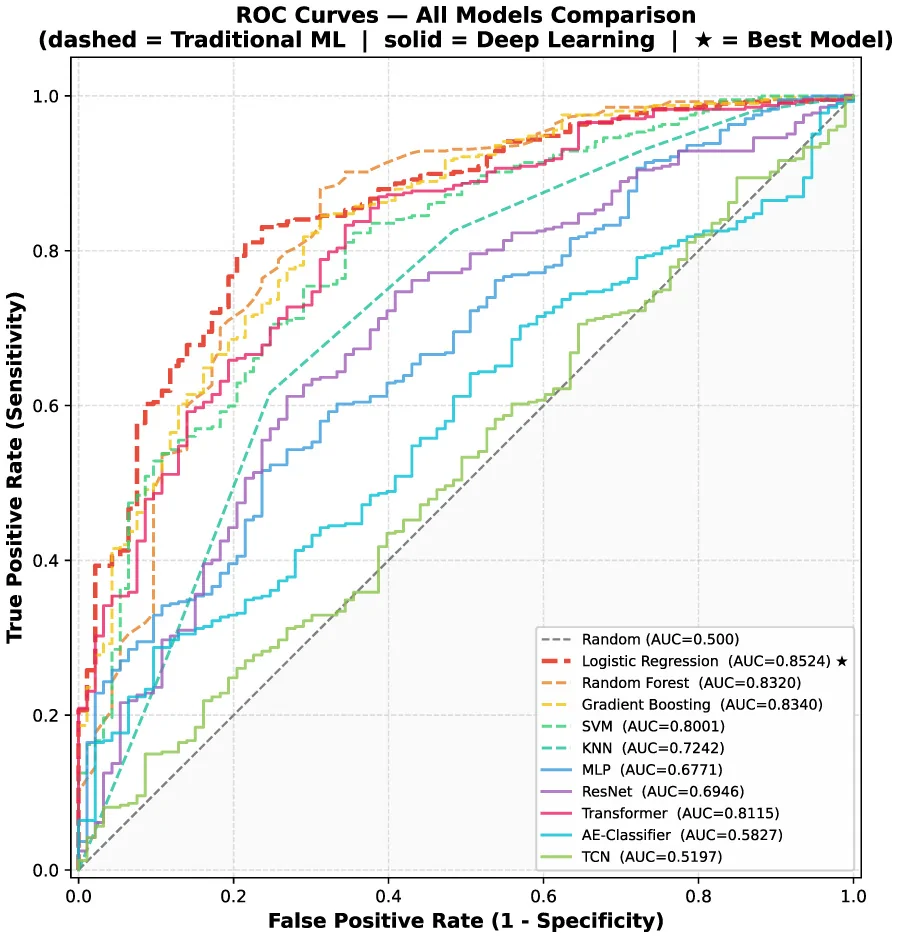
ROC curve comparison of all 10 models (dashed lines = traditional ML; solid lines = deep learning; thick red dashed line = best model, logistic regression ⋆; gray diagonal = random baseline).

**Figure 5:**
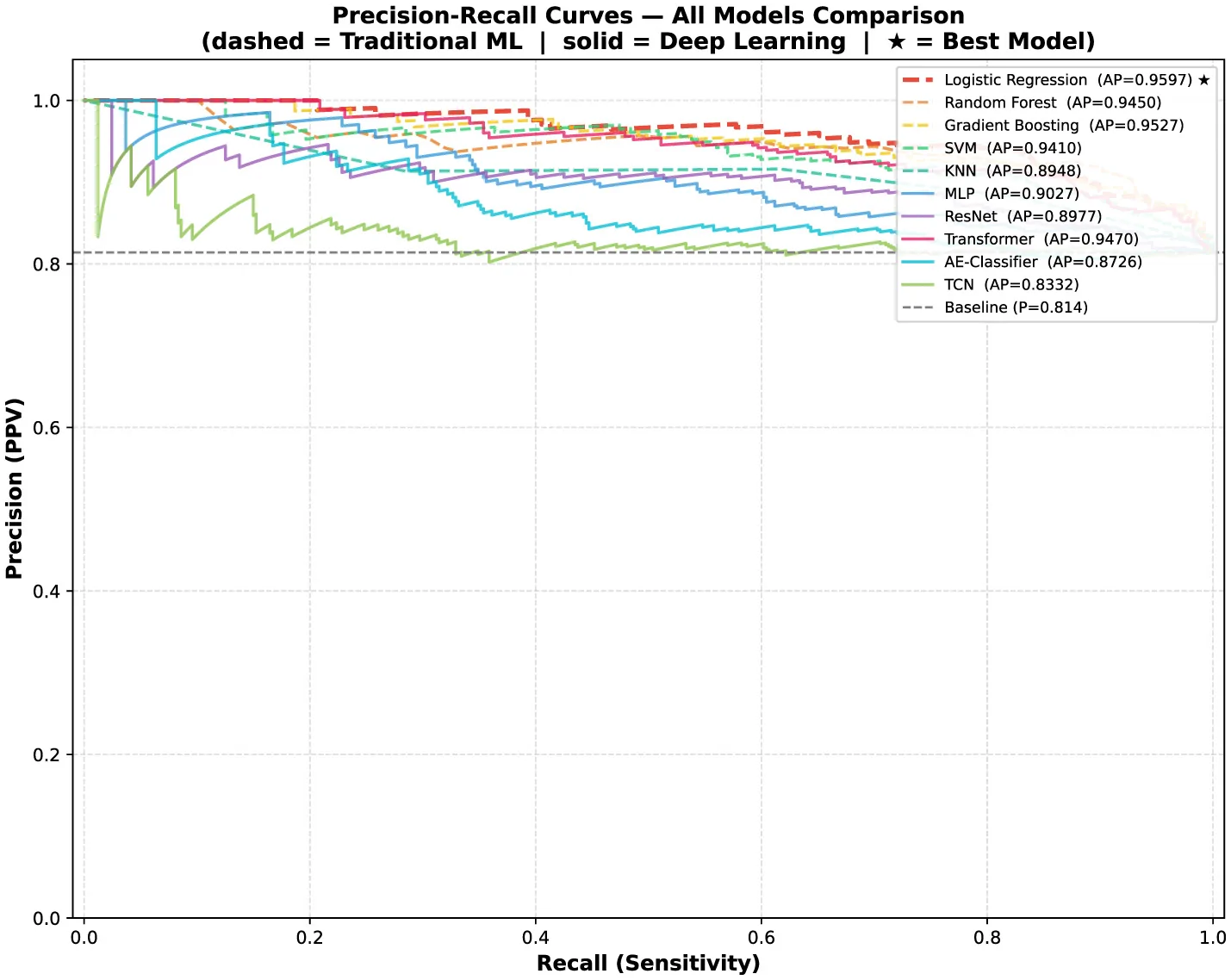
Precision-Recall curve comparison of all 10 models (dashed lines = traditional ML; solid lines = deep learning; thick red dashed line = best model, logistic regression ⋆; gray horizontal line = baseline precision *P* = 0.814).

To comprehensively compare models from two dimensions—overall classifier discriminative ability and precise predictive capability—ROC curves and PR curves were plotted separately; the results are presented in Figures 4, 5.

As shown in Figure 4, the logistic regression curve (AUC = 0.8524, ⋆) is positioned at the top of all models, and the traditional ML curve cluster (dashed lines) is overall superior to the deep learning curve cluster (solid lines). The Transformer (AUC = 0.812) is the only deep learning model whose curve overlaps with the main region of traditional ML, whereas TCN (AUC = 0.520) and AE-Classifier (AUC = 0.583) curves are close to the random diagonal, possessing almost no discriminative capability.

As shown in Figure 5, in the class-imbalanced scenario (positive rate 81.4%), logistic regression (AP = 0.9597) maintained the highest precision-recall balance across the full recall range, sustaining a precision of approximately 0.97 in the high-sensitivity region where recall >0.6. The Transformer (AP = 0.9470) performed best among deep learning models, while the TCN (AP = 0.8332) PR curve rapidly dropped to near the baseline in the moderate recall interval, confirming that its high recall is entirely driven by a strategy of predicting all samples as positive.

### 4.5 Radar chart multi-dimensional comprehensive performance comparison

**Figure 6:**
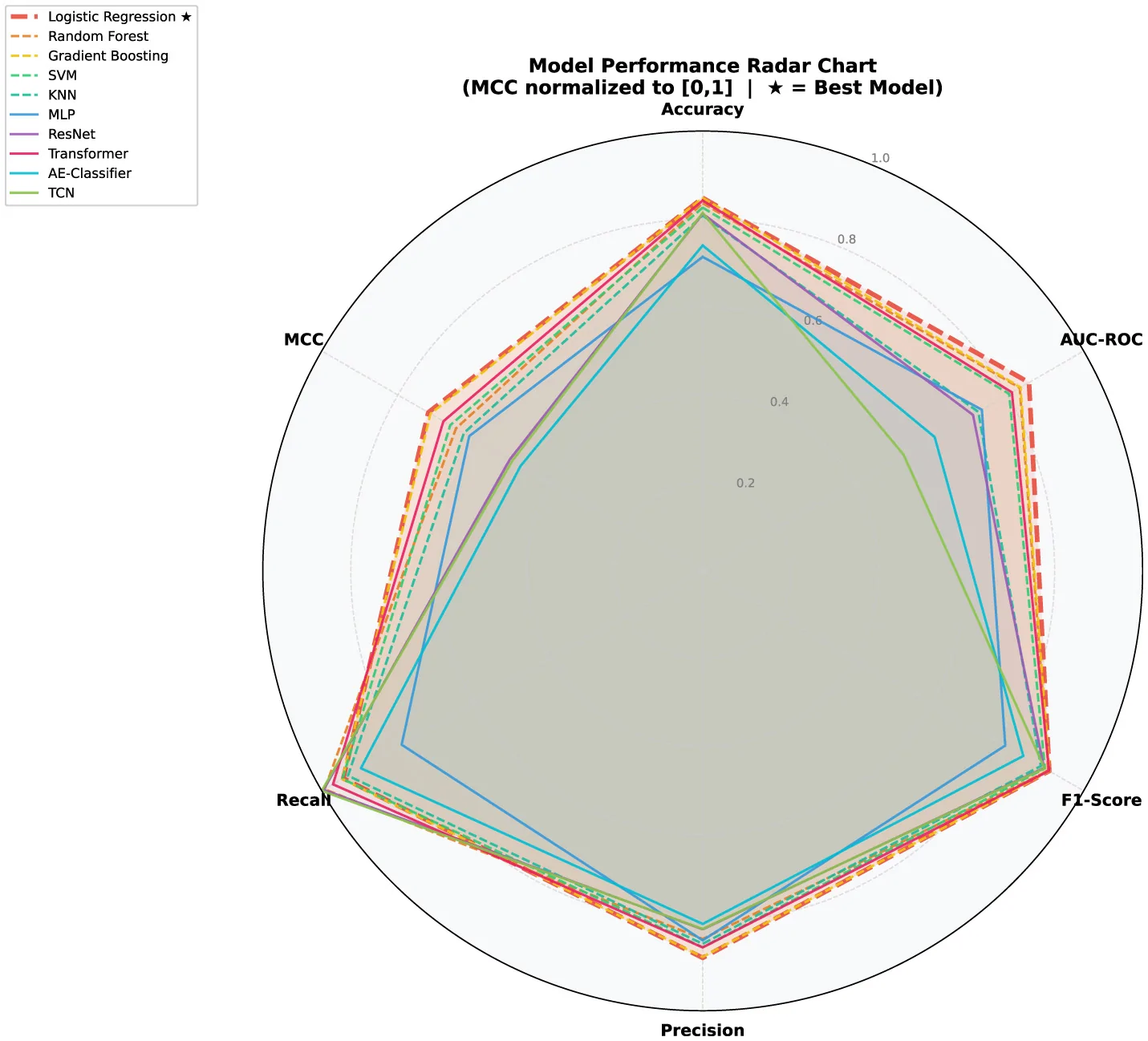
Comprehensive performance radar chart of 10 models (6 axes correspond to 6 evaluation metrics; MCC normalized to [0, 1]; dashed lines = traditional ML, solid lines = deep learning; ⋆ = best model).

To simultaneously present the overall performance profiles of 10 models across 6 metrics within a single visualization, a radar chart was plotted; the result is shown in Figure 6.

As shown in Figure 6, logistic regression (thick red dashed line) has the most prominent vertices on the Accuracy, AUC-ROC, and MCC axes, forming the largest and most balanced polygon, intuitively confirming its overall superiority. The traditional ML model cluster expands uniformly to the outer region. The Transformer most closely approaches the traditional ML cluster contour among deep learning models and is the only deep learning model without obvious axial collapse. The extremely low values of ResNet, TCN, and AE-Classifier on the MCC axis lead to severe concavity of the polygon in this dimension, fully corresponding to the class-bias phenomena observed in their confusion matrices.

### 4.6 Refined confusion matrix analysis

**Figure 7:**
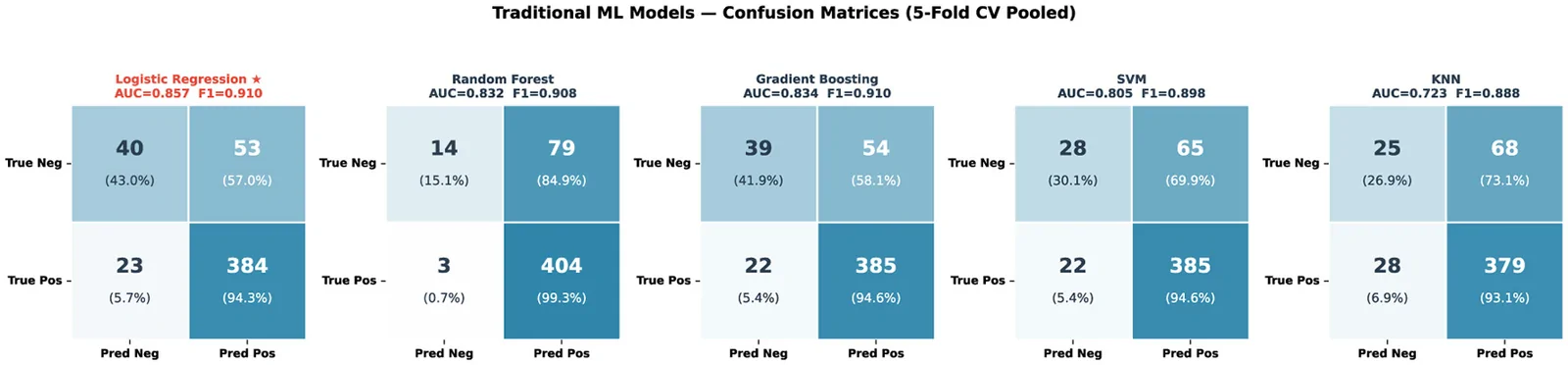
Confusion matrices of traditional machine learning models (5-fold cross-validation pooled, 500 cases in total). Color intensity represents the normalized proportion; values are absolute frequencies with row percentages in parentheses; red title = best model.

**Figure 8:**
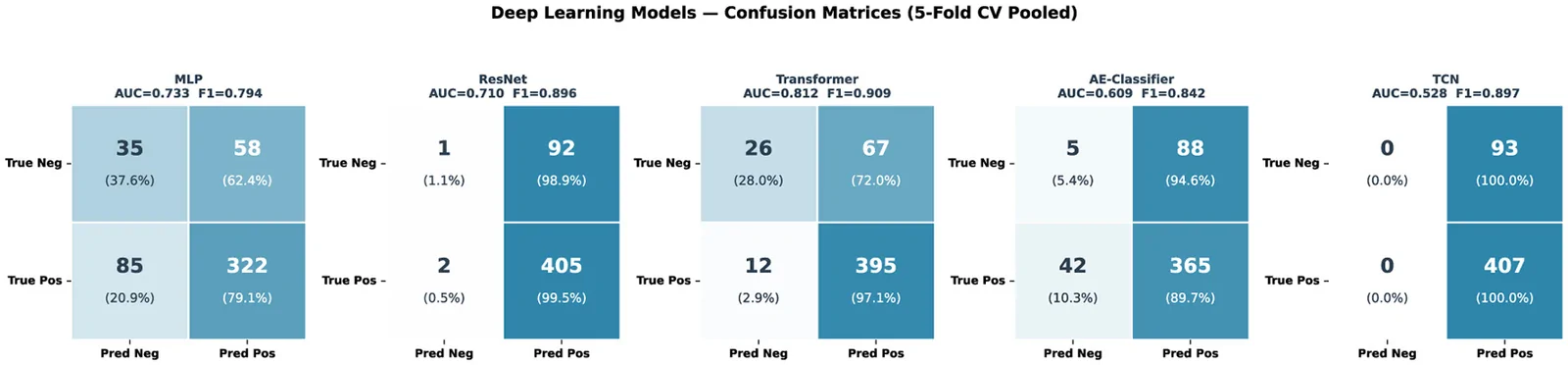
Confusion matrices of deep learning models (5-fold cross-validation pooled, 500 cases in total). Color intensity represents the normalized proportion; values are absolute frequencies with row percentages in parentheses.

To reveal the fine-grained classification behavior of each model underlying the summary metrics, confusion matrices for traditional ML (Figure 7) and deep learning (Figure 8) models were presented separately.

As shown in Figure 7, logistic regression (⋆) achieved the best balance between TN recognition rate (43.0%, 40/93) and TP recognition rate (94.3%, 384/407), demonstrating robustness under class-imbalanced conditions. The classification pattern of gradient boosting was similar to that of logistic regression (TN = 39, 41.9%; TP = 385, 94.6%). Random forest had an extremely high false positive rate (FP = 79, 84.9%); although its TP rate was near-perfect (404, 99.3%), it misclassified a large number of negative samples as high-risk, which would produce a significant excessive examination burden in clinical applications.

As shown in Figure 8, TCN exhibited extreme class collapse: TN = 0 (0%), with all 93 negative samples predicted as positive. Its AUC = 0.528 and MCC = 0.000 are indistinguishable from random guessing, and its apparent F1 = 0.897 is entirely driven by the 81.4% positive sample proportion. ResNet showed a similar pattern (TN = 1, 1.1%). The Transformer was the most balanced in the deep learning group (TN = 26, 28.0%; TP = 395, 97.1%). MLP had the highest TN recognition rate (37.6%) in the deep learning group and learned meaningful feature representations; it was therefore selected as the target model for SHAP analysis.

### 4.7 SHAP explainability analysis

#### 4.7.1 Global feature importance

**Figure 9:**
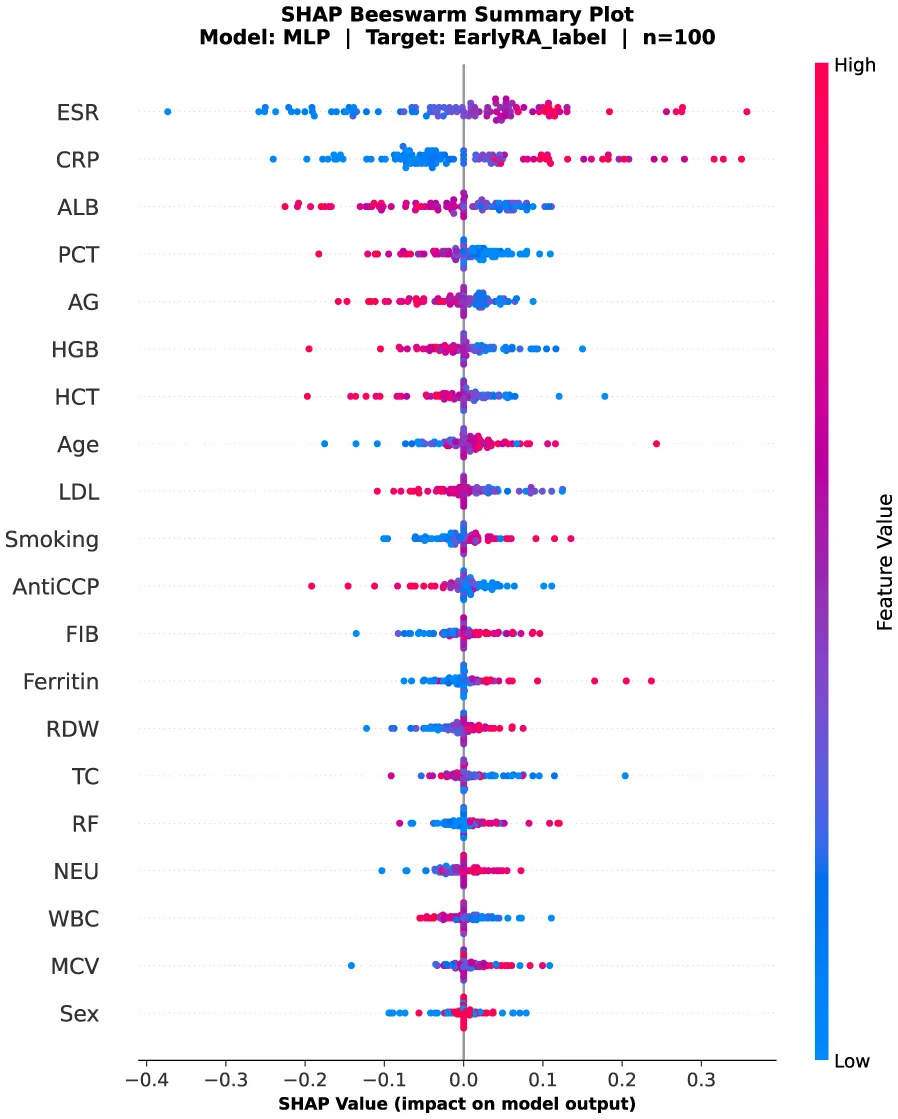
SHAP beeswarm summary plot. Features are ranked from top to bottom by mean |SHAP|; red = high feature value, blue = low feature value; *n* = 100 test samples; Model: MLP.

**Figure 10:**
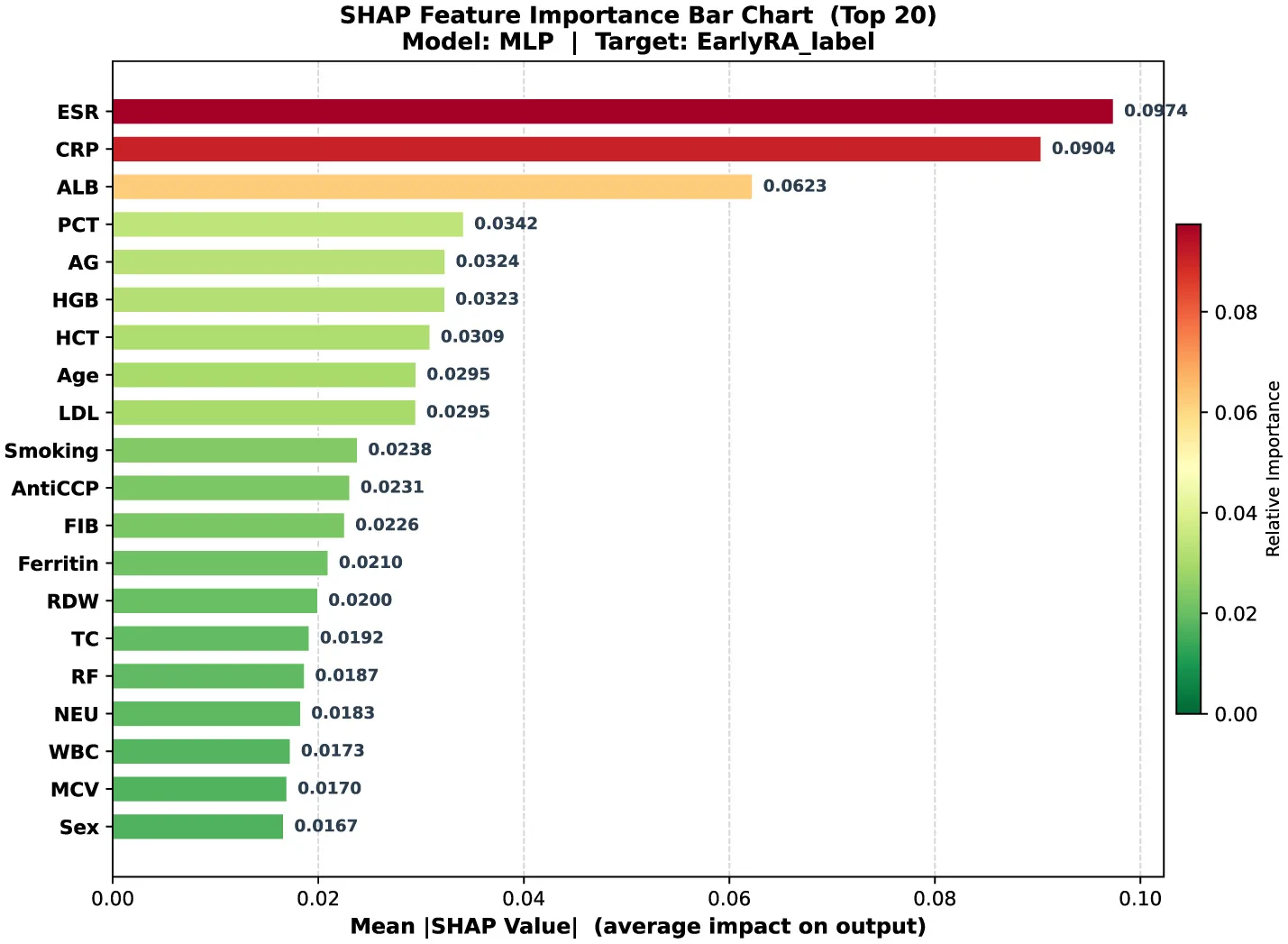
SHAP feature importance bar chart (Top 20). The horizontal axis represents the mean absolute SHAP value; the color gradient from deep red to deep green encodes relative importance.

To reveal the key driving features of the MLP model for predicting early RA onset from a global perspective, a SHAP beeswarm summary plot (Figure 9) and a feature importance bar chart (Figure 10) were generated.

As shown in Figure 9, ESR ranked first in importance (SHAP value range approximately −0.4 to +0.35); high ESR values (red dot cluster) were concentrated in the positive SHAP region while low ESR values (blue dot cluster) were distributed in the negative SHAP region, presenting a clear positive driving pattern. CRP (2nd place) showed a highly similar positive pattern to ESR. ALB (3rd place) exhibited a distinct inverse pattern: high ALB values corresponded to negative SHAP contributions and is the only protective factor among the top 6 features. PCT, AG, and HGB also displayed negative driving patterns, indicating the influences of infection-inflammation status differentiation, globulin elevation-related immune activation, and anemia of chronic disease on RA onset risk, respectively.

As shown in Figure 10, the importance of ESR (0.0974) and CRP (0.0904) far exceeded that of the third-ranked ALB (0.0623); together, these three constitute the core biomarker triad for early RA prediction, with a cumulative contribution of approximately 38% of the total feature importance. Among the remaining hematological indicators, RDW ranked 13th (Mean|SHAP| = 0.0200) in the global importance ordering of Figure 10, indicating that although emerging evidence has linked RDW to chronic inflammatory and autoimmune diseases, its incremental information appears to be partially absorbed by the more directly relevant erythrocyte indices HGB (rank 6) and HCT (rank 7) within the multi-feature joint prediction framework. Notably, Anti-CCP antibody value (0.0231) ranked only 11th, below several routine hematological indicators, indicating that within the multi-feature joint prediction framework, the combined information content of inflammatory and metabolic indicators is no less than that of the traditional single autoantibody marker. To further corroborate the protective role of albumin identified by SHAP, an exploratory univariate ROC analysis was performed using ALB alone as the predictor of EarlyRA onset, yielding AUC = 0.72 (95% CI: 0.66–0.78), confirming that albumin retains independent discriminative value even outside the multivariate framework.

#### 4.7.2 Non-linear effects and feature interactions

**Figure 11:**
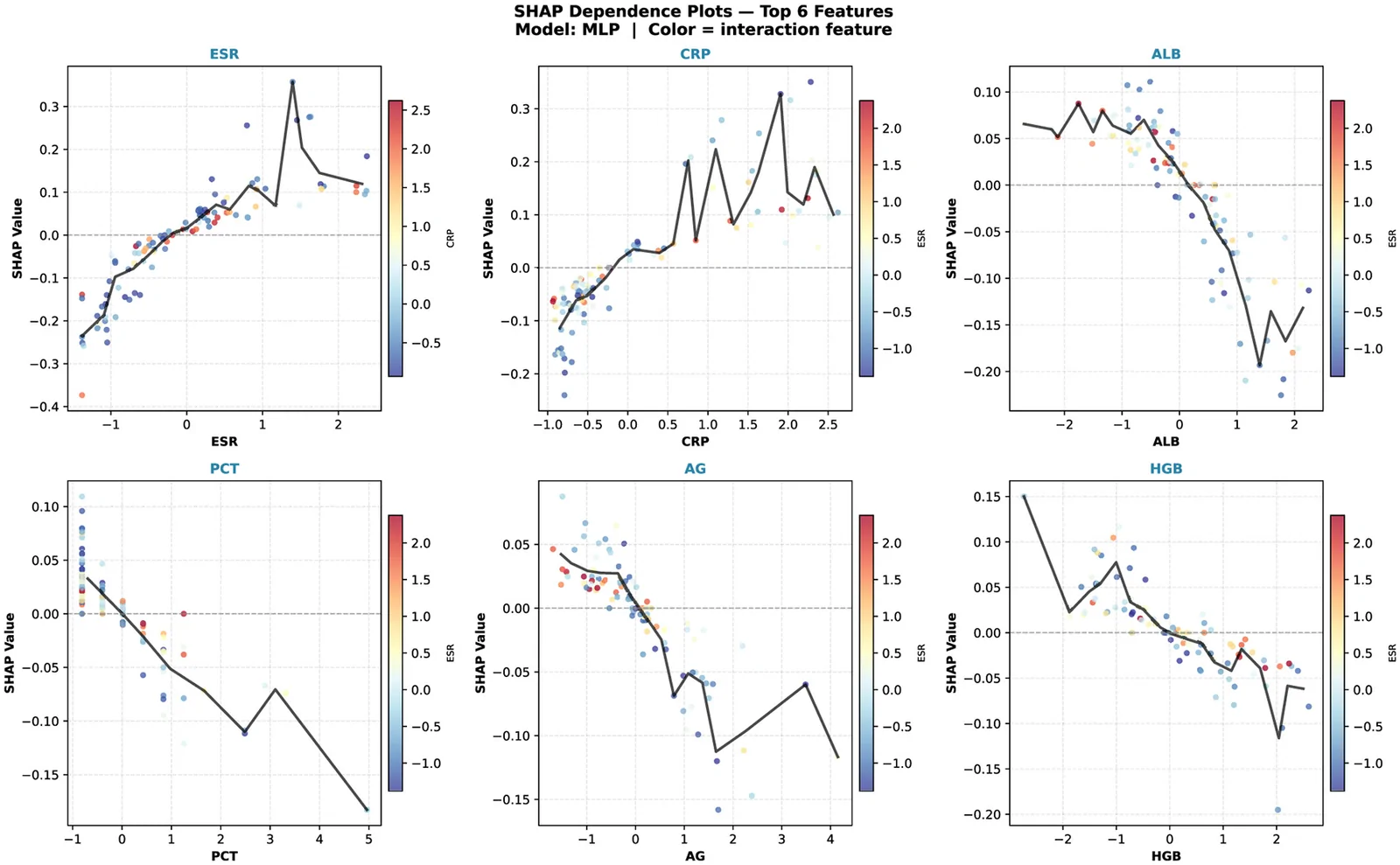
SHAP dependence plots for the top 6 features. Dot color = standardized value of the interaction variable ESR (CRP for the ESR subplot); black line = binned mean trend line; red = high, blue = low.

To further reveal the non-linear patterns of key features on prediction outcomes and the interaction effects among features, SHAP dependence plots for the top 6 features were generated, as shown in Figure 11.

As shown in Figure 11, the ESR trend line (upper left) showed a non-linear positive correlation: when standardized values rose from −1 to +1.5, SHAP values jumped from approximately −0.3 to +0.35, then plateaued beyond +1.5, displaying a clear saturation threshold effect. High CRP values clustered in the high ESR region, suggesting a positive synergistic interaction between ESR and CRP. CRP (upper middle) showed a clear activation inflection point at a standardized value of approximately +0.5, with SHAP near zero below this value. ALB (upper right) showed a monotonically decreasing negative correlation, with the zero crossing at approximately +0.5, indicating that RA risk significantly increases when ALB falls below the normal median, consistent with the pathological mechanism by which chronic inflammation reduces synthesis of the negative acute-phase protein ALB. PCT (lower left) showed a monotonically decreasing trend, reflecting the differential diagnostic significance of elevated PCT in distinguishing acute bacterial infection from RA-related autoimmune inflammation. AG ratio (lower middle) showed a monotonically decreasing trend; low AG ratio (dominated by elevated globulin) corresponded to positive SHAP contributions, consistent with the chronic immune activation pattern in RA. HGB (lower right) showed a negative correlation trend, revealing the positive association between low hemoglobin (anemia) and RA onset risk, reflecting the clinical marker value of anemia of chronic disease.

#### 4.7.3 Individualized prediction interpretation

**Figure 12:**
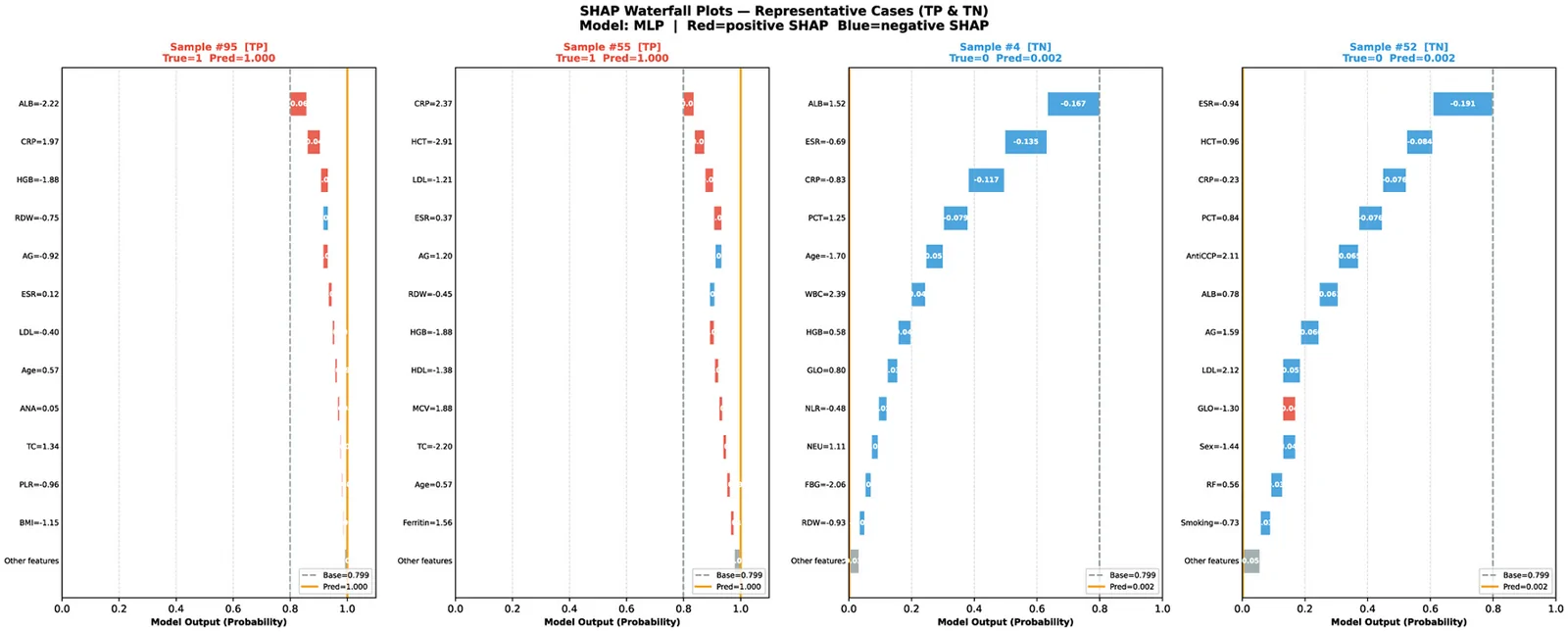
SHAP waterfall plots for representative true-positive and true-negative cases. Red bars = positive SHAP contribution; blue bars = negative SHAP contribution; orange line = final predicted probability; dashed line = baseline probability 0.799; Model: MLP.

To verify whether the model's prediction mechanism at the individual level was consistent with clinical logic, SHAP waterfall plot analysis was performed for 4 representative cases (2 TP and 2 TN), as shown in Figure 12.

As shown in Figure 12, all cases were decomposed step-by-step from the baseline probability of 0.799. **TP Sample #95** (Pred = 1.000): low ALB (−2.22, SHAP = +0.060) and high CRP (+1.97, SHAP = +0.047) jointly drove the prediction, combined with low HGB (+0.028) and mildly elevated ESR (+0.014), pushing the predicted probability to 1.000 through multi-factor synergy. **TP Sample #55** (Pred = 1.000): high CRP (+2.37, SHAP = +0.038) combined with extremely low HCT (−2.91, SHAP = +0.037) constituted the main driving force, indicating that the co-occurrence of acute inflammation and significant anemia is an important signal combination for high-confidence RA prediction. **TN Sample #4** (Pred = 0.002): high ALB (+1.52, SHAP = −0.167) contributed the strongest protective effect; low ESR (−0.135) and low CRP (−0.117) cooperatively reduced the probability to 0.002. **TN Sample #52** (Pred = 0.002): low ESR (−0.94, SHAP = −0.191) contributed the largest single protective effect. Notably, this case had an elevated Anti-CCP value (+2.11) but its SHAP contribution was still negative (−0.065), indicating that a single elevated Anti-CCP value is insufficient to dominate the prediction direction against a background of multiple low inflammatory indicators, demonstrating the advantage of multi-feature integrated prediction over single-marker threshold judgement. The SHAP decomposition results of all 4 cases were highly consistent with clinical pathological logic, confirming that the model's prediction mechanism has good clinical interpretability.

### 4.8 Model calibration analysis

**Figure 13:**
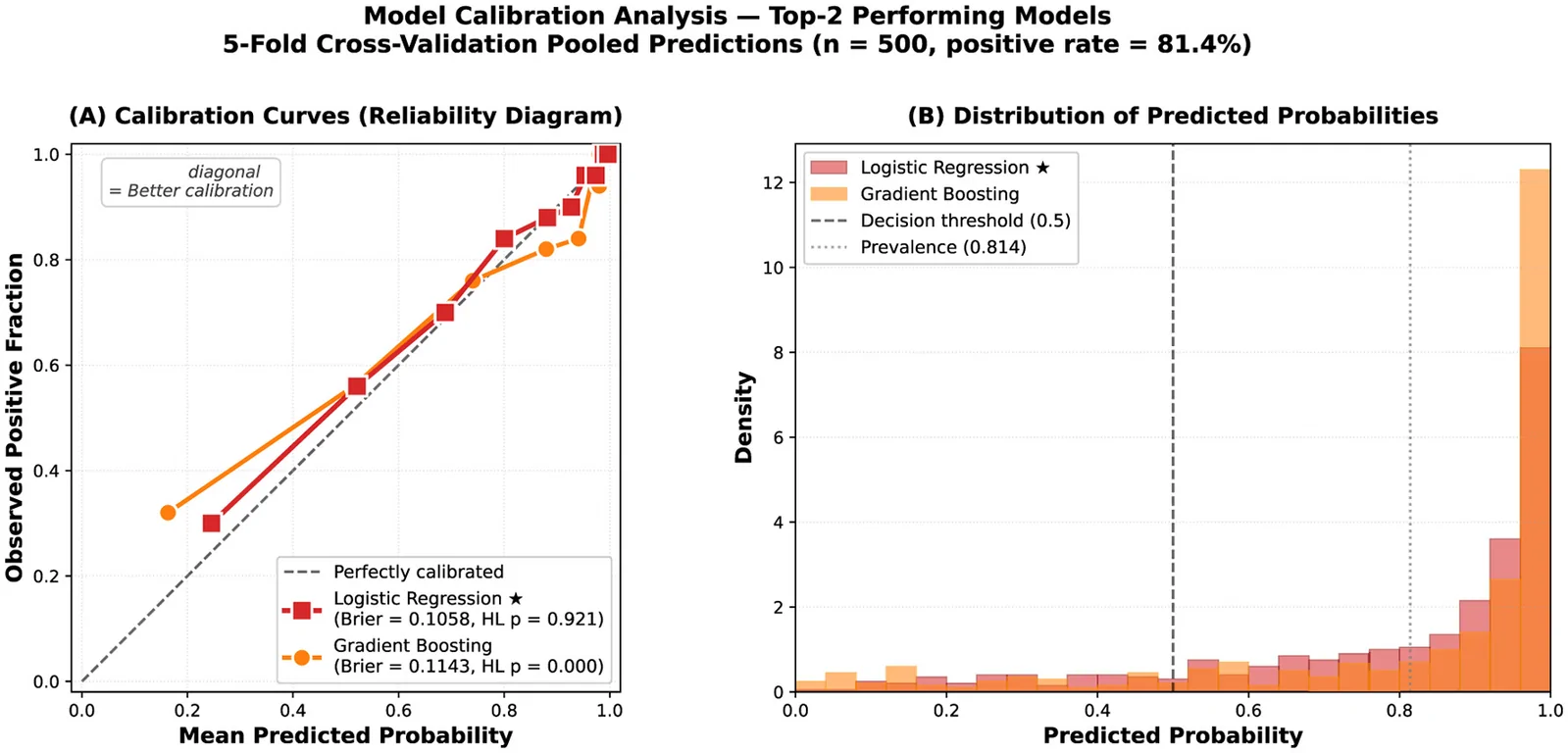
Model calibration analysis of the top-2 performing models. **(A)** Calibration curves (reliability diagram) using 10-quantile binning; logistic regression (red squares, ⋆) closely tracks the diagonal of perfect calibration (Brier = 0.106, HL χ^2^= 3.20, *p*= 0.921), while gradient boosting (orange circles) shows mild over-confidence at the probability extremes (Brier = 0.114, HL χ^2^= 30.7, *p* < 0.001). **(B)** Distribution of predicted probabilities; both models concentrate mass above the population prevalence (0.814), consistent with the high RA-positive rate of the cohort.

Beyond discriminative metrics, calibration determines whether the predicted probabilities can be interpreted as faithful risk estimates—an essential requirement for any clinical decision-support tool. We therefore evaluated the calibration of the two best-performing models (logistic regression and gradient boosting) using reliability diagrams, Brier scores, and the Hosmer–Lemeshow χ^2^ test, with the predicted probabilities pooled from the 5-fold cross-validation procedure described in Section 3.4. The results are presented in Figure 13.

As shown in Figure 13A, logistic regression yielded the lowest Brier score (0.106, far below the non-informative baseline of 0.154) and the highest Hosmer–Lemeshow *p*-value (0.921, well above the conventional threshold of 0.05), indicating that its predicted probabilities are statistically indistinguishable from observed event frequencies—i.e., the model is well calibrated. Gradient boosting, while achieving comparable discrimination (AUC = 0.834), showed a mild but statistically detectable miscalibration pattern (Brier = 0.114, HL *p* < 0.001), with predicted probabilities systematically shifted toward the extremes of 0 and 1; this is a well-documented behavior of tree-based boosting algorithms whose decision-tree leaves sharpen the output distribution. Figure 13B further shows that both models concentrate the probability mass above 0.5, mirroring the high positive prevalence of the cohort. Taken together, the calibration analysis confirms that logistic regression provides not only the best discrimination but also the most reliable probabilistic risk estimates among the candidate models, supporting its suitability as the recommended deployment choice for early RA risk stratification in routine clinical practice.

## 5 Discussion

### 5.1 Performance advantages of traditional machine learning on small-sample structured medical data

One of the most central findings of this study is that, on a structured laboratory dataset of 500 samples, traditional machine learning models were overall superior to deep learning models, with logistic regression achieving the best composite performance (AUC = 0.857, MCC = 0.441). This result is highly consistent with the conclusions of several recent systematic studies targeting structured clinical tabular data. Greener et al. (11) noted that the performance of machine learning models is highly dependent on the match between data scale and feature dimensionality, and that in low-dimensional structured data, parsimonious linear models tend to outperform overparameterised deep networks. The meta-analysis by Huang et al. (7) likewise demonstrated that traditional machine learning models exhibit stronger generalization robustness in real-world medical datasets with limited sample sizes. From an algorithmic mechanism perspective, logistic regression effectively controlled the risk of overfitting through L2 regularization (40), and its linear decision boundary precisely matched the inherently separable structure of the data in this study; gradient boosting (41) captured non-linear interactions among inflammatory indicators such as ESR and CRP through sequential residual correction, achieving the second-best performance.

In contrast, ResNet (12) and TCN (14) exhibited severe class collapse—predicting almost all samples as positive, with MCC approaching 0. This phenomenon should be interpreted with caution: rather than reflecting an inherent unsuitability of residual or convolutional architectures for RA prediction, it more parsimoniously reflects the joint effect of (i) the high class imbalance of the cohort (positive rate 81.4%), (ii) the deliberate methodological decision not to apply class weighting or resampling in any of the ten models (see Section 3.5), so as to preserve a strictly architecture-comparable evaluation, and (iii) the relatively small sample size, which limits the ability of these high-capacity networks to learn the minority-class manifold. Rahman et al. (24) and Thabtah et al. (25) both emphasized that class imbalance is one of the primary sources of inflated classifier performance in medical datasets, while Chicco and Jurman (26) demonstrated the irreplaceable advantage of MCC over the F1 score in class-imbalanced evaluation—the fact that TCN achieved an F1 as high as 0.897 but an MCC of 0.000 in this study precisely corroborates this argument. Importantly, future work should systematically reassess these architectures under explicit class-imbalance mitigation strategies (cost-sensitive loss, focal loss, SMOTE-based oversampling) before drawing any architecture-specific conclusions. The Transformer (13) performed relatively well in the deep learning group (AUC = 0.812); its feature-level self-attention mechanism effectively captured the synergistic relationships among multiple inflammatory indicators, but the attention matrix failed to fully converge due to insufficient training samples. These findings carry important methodological implications for clinical machine learning research: when constructing small-sample structured medical predictive models, traditional machine learning models should be evaluated with priority, and MCC should be reported as a mandatory core evaluation metric rather than relying solely on the superficially appealing Recall or F1 scores.

### 5.2 Clinical pathological mechanism interpretation of core biomarkers

The ESR–CRP–ALB core biomarker triad revealed by SHAP analysis has a solid pathophysiological foundation in rheumatology and represents the most important clinical translational value of this study. ESR (Mean|SHAP| = 0.097) and CRP (0.090), as the most important positive predictive drivers, are core components of the acute-phase reactant score in the 2010 ACR/EULAR RA classification criteria (39). Although both reflect systemic inflammatory burden, their mechanisms differ: CRP is a positive acute-phase protein synthesized by the liver under IL-6 drive and is sensitive to fluctuations in acute inflammation, whereas ESR is influenced by multiple proteins including fibrinogen and globulins and is more robust for continuous monitoring of chronic low-grade inflammation. The positive synergistic interaction between ESR and CRP revealed by the SHAP dependence plots indicates that their simultaneous elevation has a supra-additive synergistic promoting effect on RA prediction, which is highly consistent with the clinical practice of jointly applying ESR and CRP in disease activity scores such as DAS28. Salem et al. (42) and Scher et al. (43) confirmed the core driving role of systemic inflammation in RA pathogenesis from the perspectives of gut microbiome and animal arthritis models, respectively; consistent with the state-of-the-art review by Romão and Fonseca (1), which summarized inflammation and immune dysregulation as the central pathobiological mechanisms underlying RA onset, and with Sagris et al. (44) on the role of chronic low-grade inflammation in the progression of autoimmune diseases. Together, these independent biological lines of evidence support the high SHAP importance of ESR and CRP reported in this study. The finding that ALB is the most important protective factor (Mean|SHAP| = 0.062, negative direction) is highly consistent with the “reverse acute-phase protein effect” in RA—pro-inflammatory cytokines such as IL-6 and TNF-α drive the liver to preferentially synthesize positive acute-phase proteins while simultaneously suppressing albumin synthesis, leading to a sustained decline in serum ALB levels. This temporal cascade—in which sustained IL-6-driven elevation of CRP precedes and contributes to the gradual decline in serum ALB—implies that albumin reduction lags behind, rather than precedes, inflammatory marker elevation, which is also consistent with the univariate ROC analysis of ALB alone (AUC = 0.72, 95% CI: 0.66–0.78) reported in Section 4.7.1: although albumin retains independent discriminative value, its predictive strength is intrinsically derived from the upstream inflammatory state captured more directly by ESR and CRP. The non-linear zero-crossing point of ALB at a standardized value of approximately +0.5 in the SHAP dependence plot provides a clinically actionable early warning threshold: RA risk increases significantly when ALB falls below the normal median, and this finding can provide auxiliary support for early RA risk stratification without any additional testing cost. It is noteworthy that the Anti-CCP antibody value (0.0231) ranked only 11th in global importance; this does not negate its clinical diagnostic status, but rather reveals an important methodological finding: within a multi-feature joint prediction framework, the combined information content of routine inflammatory and metabolic indicators is no less than that of the traditional single gold-standard biomarker (21, 27), suggesting that clinicians should fully appreciate the systematic value of routine hematological indicators in early RA risk stratification while also attending to specific markers.

### 5.3 Clinical translational significance, calibration, and study limitations

The individualized analysis from SHAP waterfall plots revealed distinctly different feature-driving pathways underlying positive or negative predictions of the same class, which is of great significance for precision medicine practice in RA. Taking the two true-positive cases as examples: the prediction of Sample #95 was mainly driven by low ALB (SHAP = +0.060), suggesting a chronic inflammatory phenotype dominated by protein consumption; whereas Sample #55 was characterized by high CRP combined with extremely low HCT as the core signals (SHAP = +0.038 and +0.037, respectively), suggesting a phenotype of acute inflammation accompanied by significant anemia. Both patients were correctly identified as high-risk, but their biological signal pathways were entirely different—this is precisely the core advantage of SHAP individualized interpretation over traditional population statistics. Ma et al. (20) and El-Sappagh et al. (22) likewise observed the critical supporting value of SHAP individual-level interpretation for personalized clinical decision-making in cardiovascular and neurological disease prediction. In the true-negative case Sample #52, the Anti-CCP value was elevated (+2.11) but its SHAP contribution remained negative (−0.065), indicating that a single elevated Anti-CCP value alone is insufficient to dominate the prediction direction against a background of multiple low inflammatory indicators, demonstrating the overall advantage of multi-feature integrated prediction over single-marker threshold judgement—a finding that mutually corroborates the conclusion of Yuan et al. (27) that multidimensional ensemble modeling outperforms single biomarkers in cardiovascular risk prediction.

#### 5.3.1 Clinical positioning of the model

A subtle but important clarification concerns the temporal relationship between the baseline measurement and the formal RA diagnosis. In the present cohort, baseline blood sampling was performed at the patient's first rheumatology consultation, at which point the rheumatologist had not yet established a definitive RA diagnosis; over the 12-month follow-up, some patients were subsequently classified as RA according to the 2010 ACR/EULAR criteria (39), while others received alternative diagnoses such as osteoarthritis, fibromyalgia, or undifferentiated arthritis. The proposed model should therefore be positioned as a *first-visit risk stratification aid*—a tool that quantifies the probability of progression toward RA from routinely available laboratory data, rather than a replacement for longitudinal rheumatologist evaluation. This positioning is also consistent with the EULAR recommendations summarized by Kedra et al. (10), who identified clinical decision support at the screening stage as one of the most promising entry points for machine learning in rheumatology.

#### 5.3.2 Clinical relevance of model calibration

Beyond discrimination, the calibration analysis in Section 4.8 demonstrated that logistic regression produced well-calibrated probability estimates (Brier = 0.106, Hosmer–Lemeshow *p*= 0.921), whereas gradient boosting, despite comparable discrimination, exhibited mild over-confidence at the probability extremes (HL *p* < 0.001). For a clinical risk prediction tool, miscalibrated probabilities can mislead downstream decisions even when discrimination is acceptable—for example, an over-confident probability of 0.95 may be misinterpreted as near-certainty when the true posterior risk is in fact lower. The combined evidence of strong discrimination, robust MCC, and excellent calibration therefore supports logistic regression as the recommended deployment choice for early RA risk stratification in routine clinical practice.

However, several limitations of this study must be acknowledged. **First**, the sample size (*n* = 500) is relatively limited; deep learning models generally require larger-scale datasets to fully exploit their parameter advantages, and future studies should expand multi-center sample sizes to verify the external generalization capability of the models (28, 29). **Second**, this study adopted a single-center retrospective design, which carries a risk of institution-specific bias; no external validation in an independent cohort or geographically distinct site was performed in the present work, and multi-center prospective external validation with prospectively collected data should therefore be considered the priority task for subsequent research. **Third**, joint ultrasound and MRI imaging scores, as well as genetic susceptibility gene data such as HLA-DRB1, were not incorporated; multi-modal data fusion is expected to further improve predictive performance (38). **Fourth**, for the class collapse observed in ResNet and TCN, future research should systematically explore class imbalance handling strategies such as SMOTE oversampling and cost-sensitive learning (24, 25) to fully unleash the latent discriminative capability of deep learning architectures. **Fifth**, KernelExplainer relies on K-Means background approximation, which introduces certain precision limitations in SHAP estimation for low-importance features; DeepExplainer, specifically designed for neural networks, could be adopted in future work to improve computational accuracy (18). **Sixth**, intermittent over-the-counter NSAID use was permitted at enrolment (see Section 3.1); although systemic glucocorticoids and DMARDs were excluded, NSAIDs can modestly attenuate CRP and ESR levels and may therefore introduce limited confounding into the inflammatory feature distribution. Future studies should record cumulative NSAID dosage and perform formal sensitivity analyses to quantify this effect. **Seventh**, although the proposed SHAP framework provides population-level and individual-level interpretation, head-to-head benchmarking against RA-specific machine learning models reported in the recent literature [e.g., Norgeot et al. (8); Maarseveen et al. (9); Myasoedova et al. (16)] was not performed because these works target different prediction tasks (longitudinal disease activity, EHR-based patient identification, methotrexate response). Constructing a harmonized benchmark dataset for cross-study comparison remains an open methodological challenge that we will pursue in follow-up work. **Eighth**, low-titer and high-titer anti-CCP subgroups were not separately modeled in this binary-classification framework; future analyses with larger cohorts could explore ordinal stratification of anti-CCP titres for finer-grained risk profiling.

## 6 Conclusion

This study constructed and systematically evaluated a machine learning prediction pipeline based on 29 routine hematological laboratory parameters for binary classification of early rheumatoid arthritis onset within 12 months (with anti-CCP positivity examined as a complementary auxiliary outcome), and conducted a comprehensive analysis of the predictive decision mechanism of the best deep learning model through four-dimensional SHAP explainability analysis, together with formal calibration assessment of the top-performing models. The experimental results demonstrated that among 10 comparison models, logistic regression ranked first with the best overall performance (AUC = 0.857, F1 = 0.910, MCC = 0.441, Brier = 0.106, Hosmer–Lemeshow *p*= 0.921); traditional machine learning models were overall superior to deep learning models at the current data scale; the class collapse observed in some deep learning models (ResNet and TCN) —understood as the joint consequence of class imbalance, the deliberate exclusion of imbalance-mitigation strategies in the comparison protocol, and the limited sample size— further revealed the potential risks of applying deep learning in small-sample class-imbalanced scenarios and emphasized the irreplaceable role of MCC as a robust evaluation metric. SHAP analysis quantitatively revealed the core status of ESR (Mean|SHAP| = 0.097) and CRP (0.090) as the most important positive driving factors, and albumin ALB (0.062) as the most critical protective factor; together, these three constitute the core biomarker triad for early RA risk prediction, with a cumulative contribution of approximately 38% of the total feature predictive information. Dependence plots further revealed the positive synergistic interaction effect of ESR and CRP and the non-linear protective threshold effect of ALB; individualized decomposition from waterfall plots confirmed that the model prediction mechanism is highly consistent with clinical pathological logic, significantly enhancing the clinical credibility and interpretability of the model. The calibration analysis additionally confirmed that the recommended logistic regression model not only discriminates well but also produces probabilistic risk estimates that are statistically indistinguishable from observed event frequencies, an essential requirement for any clinical decision-support tool. This study confirms that a well-performing early RA risk prediction model can be constructed using routine clinical hematological test results alone, providing data-driven evidence-based support for the systematic integration of routine inflammatory and nutritional metabolic indicators such as ESR, CRP, and ALB into early screening practice in rheumatology and immunology, and laying an important foundation for the translational application of machine learning-assisted diagnostic tools in RA clinical management. Future work will focus on multi-center prospective external validation, multi-modal data fusion incorporating imaging and genetic susceptibility data, systematic evaluation of class-imbalance mitigation strategies for deep learning architectures, and head-to-head benchmarking against existing RA-specific machine learning frameworks to consolidate the clinical translational value of the proposed pipeline.

## Data Availability

The raw data supporting the conclusions of this article will be made available by the authors, without undue reservation.
