# Supplementary material for: Predicting anti-CCP positivity and early rheumatoid arthritis onset from routine laboratory parameters: a SHAP-explained machine learning pipeline
Source: Front Med (Lausanne). 2026 Jun 29;13:1834291. doi: 10.3389/fmed.2026.1834291 (PMC13447827; doi:10.3389/fmed.2026.1834291)
Supplement: Supplementary file 1 [file Supplementary_File_1.pdf]

Supplementary Material

Predicting anti-CCP positivity and early rheumatoid arthritis onset from routine laboratory parameters: a SHAP-explained machine learning pipeline

This file contains the supplementary items referenced in the main text: the deep-learning training diagnostics (Figure S1, cited in Section 3.5.2) and the logistic regression coefficient ranking (Table S1, cited in Section 3.8). Both were generated from the same dataset (n = 500), feature set (29 routine laboratory parameters), and preprocessing (fold-wise / split-wise z-score standardisation) described in the Methods, with the primary outcome EarlyRA_label.

S1. Training diagnostics of the deep learning models

Training and validation binary cross-entropy (BCE) loss were monitored for the three non-collapsing deep learning architectures (MLP, ResNet, Transformer) on an 80/20 stratified train/validation split of EarlyRA_label. Early stopping (patience = 12, restore_best_weights = True) on the validation loss was applied, consistent with the Methods. In all three models the training loss decreased monotonically and the validation loss reached its minimum within the first ~10 epochs, triggering early stopping well before the 50-epoch mark; the restored best epoch is marked on each panel. The subsequent rise in validation loss reflects the expected overfitting of high-capacity networks on a small structured cohort and motivates the early-stopping strategy adopted throughout.

Figure S1. Training and validation loss curves for the deep learning models (80/20 split, EarlyRA_label)

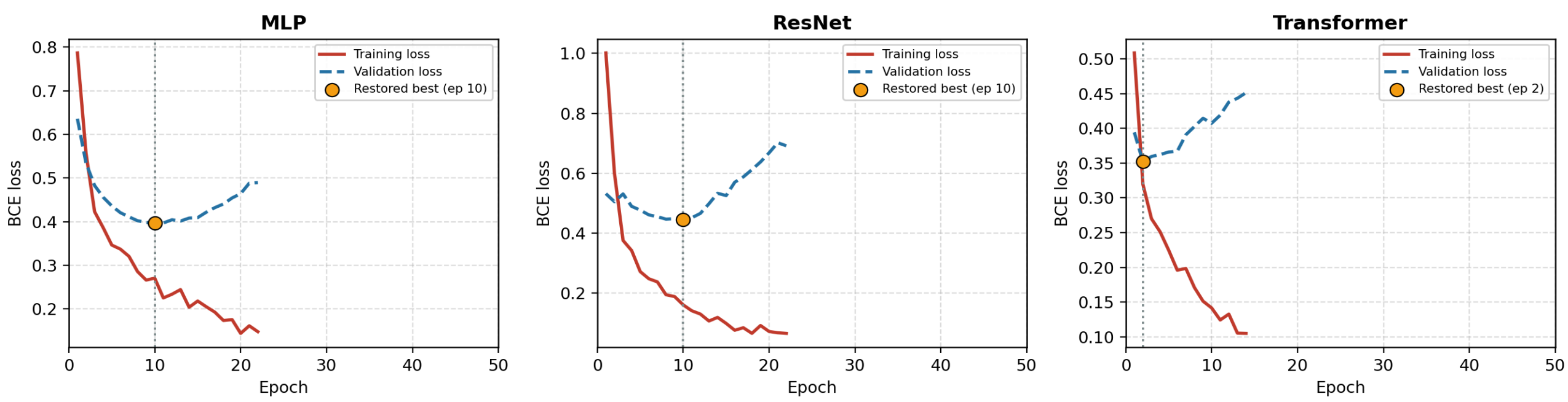

Figure S1. Training (solid red) and validation (dashed blue) BCE loss versus epoch for MLP, ResNet and Transformer. Dotted grey line and orange marker indicate the epoch at which the best validation loss occurred and weights were restored. Training was halted by early stopping (patience = 12) in every case, confirming convergence before the 50th epoch.

S2. Logistic regression coefficient ranking

To corroborate the SHAP-based feature importance obtained for the MLP, the standardised coefficients of the best-performing overall model (logistic regression with L2 regularisation) were ranked by absolute magnitude. Features were z-score standardised prior to fitting, so the coefficients are directly comparable. The three top-ranked features are CRP, ESR and ALB — the same core biomarker triad identified by the MLP-based SHAP analysis (ESR, CRP, ALB) — with ALB carrying a negative coefficient (odds ratio < 1), consistent with its role as a protective factor. This agreement between an intrinsically interpretable linear model and the post-hoc SHAP explanation supports the robustness of the reported biomarker triad across model types.

Rank	Feature	Coefficient	|Coefficient|	Odds ratio
**1**	**CRP**	**1.4543**	**1.4543**	**4.2813**
**2**	**ESR**	**1.1886**	**1.1886**	**3.2826**
**3**	**ALB**	**-0.8279**	**0.8279**	**0.4370**
4	Smoking	0.4657	0.4657	1.5931
5	GLO	0.4634	0.4634	1.5894
6	HCT	-0.4112	0.4112	0.6629
7	RDW	0.3252	0.3252	1.3843
8	AG	0.2981	0.2981	1.3473
9	LDL	-0.2930	0.2930	0.7461
10	Age	0.2751	0.2751	1.3167
11	HGB	-0.2240	0.2240	0.7993
12	Ferritin	0.2102	0.2102	1.2339
13	FBG	0.1612	0.1612	1.1749
14	TC	-0.1497	0.1497	0.8610
15	UA	-0.1336	0.1336	0.8749
16	BMI	-0.1311	0.1311	0.8771
17	RF	0.1305	0.1305	1.1394
18	PCT	-0.1276	0.1276	0.8802
19	AntiCCP	0.1244	0.1244	1.1325
20	MCV	-0.1099	0.1099	0.8960
21	ANA	0.0974	0.0974	1.1023
22	PLR	0.0973	0.0973	1.1021
23	NEU	0.0935	0.0935	1.0980
24	LYM	-0.0701	0.0701	0.9323
25	HDL	0.0533	0.0533	1.0547
26	NLR	-0.0468	0.0468	0.9543
27	FIB	0.0340	0.0340	1.0346
28	Sex	0.0259	0.0259	1.0263
29	WBC	-0.0122	0.0122	0.9878

Table S1. Standardised logistic regression coefficients for all 29 features (primary outcome EarlyRA_label), ranked by absolute coefficient. Positive coefficients (odds ratio > 1) increase predicted RA risk; negative coefficients (odds ratio < 1) are protective. The top three features (highlighted) coincide with the ESR–CRP–ALB triad from the SHAP analysis.
